# Chirality Transfer to the Magnetic Sublattice in the Hybrid Perovskite (R)-/(S)-3-Fluoropyrrolidinium Copper(II) Chloride

**DOI:** 10.1021/jacs.6c11636

**Published:** 2026-07-31

**Authors:** Zheng Zhang, Mingyu Xu, Jose L. Gonzalez Jimenez, Stephen Zhang, Weiwei Xie, Xianghan Xu, Daniel B. Straus

**Affiliations:** † Department of Chemistry, 5783 Tulane University, New Orleans, Louisiana 70118, United States; ‡ Department of Chemistry, 3078 Michigan State University, East Lansing, Michigan 48824, United States; § Department of Chemistry, 6740 Princeton University, Princeton, New Jersey 08540, United States; ∥ School of Physics and Astronomy, University of Minnesota, Twin Cities, Minnesota 55455, United States

## Abstract

Incorporating chiral organic cations into organic–inorganic hybrid materials has been shown to enable the inorganic sublattice to display chiroptical properties. We report a new two-dimensional magnetic (S = 1/2) chiral metal halide perovskite, (R)- and (S)-(C_4_H_9_FN)_2_CuCl_4_ (where (C_4_H_9_FN)^+^ is 3-fluoropyrrolidinium), which consists of Cu–Cl inorganic layers separated by (C_4_H_9_FN)^+^ organic cations. The presence of the chiral (C_4_H_9_FN)^+^ organic cation induces the formation of chiral magnetic order, even though the inorganic sublattice itself is nearly structurally centrosymmetric. We also report the racemic variant, containing an equal amount of (R)- and (S)- cations, which shows no evidence of chiral magnetic order. When the magnetic susceptibility is measured perpendicular to the inorganic Cu–Cl layer propagation direction, an antiferromagnetic phase transition at Néel temperature *T_N_* = 2.23 K is observed in both the chiral and racemic materials, and the existence of the magnetic phase transition is supported by specific heat capacity measurements. Field-induced magnetic chirality is observed through the existence of a second-order magnetoelectric effect in the chiral variant, while no magnetoelectric signal is observed for the racemic material, indicating the absence of magnetic chirality. Our findings demonstrate that materials exhibiting chiral magnetic order can be created through the incorporation of a chiral cation into an organic–inorganic hybrid magnetic material, potentially allowing for the design of tailored materials that combine chiral magnetism with other desirable optical and electronic properties that come from structural chirality.

## Graphical abstract

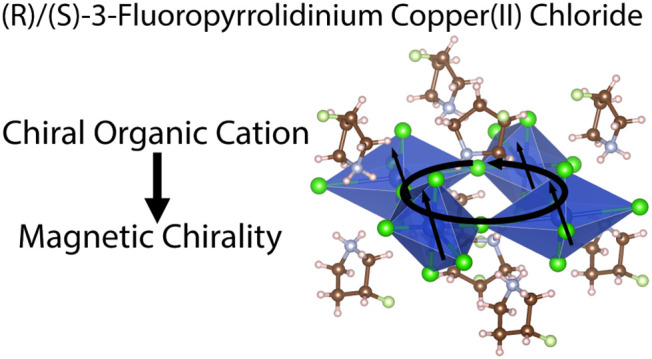

## 1 Introduction

Chiral magnetic semiconductors that demonstrate fascinating chiral, semiconducting, and magnetic properties are highly sought after for practical applications such as new forms of high-speed nonvolatile memory and chiral magnetic field sensors.^1–4^ For a material to exhibit magnetic chirality, it must have chiral spin-ordered states and/or chiral spin textures.^5,6^ Structurally chiral magnetic materials are not guaranteed to exhibit chiral magnetic ground states, with an example being Ni_3_TeO_6_.^5,7^ Magnetic chirality has been observed in achiral materials including YMn_6_Sn_6_,^8^ further demonstrating that structural and magnetic chirality are not always connected.^8–11^ In the structurally chiral inorganic materials Ba_3_NbFe_3_Si_2_O_14_,^12^ Cr_1/3_TaS_2_,^13^ and doped Ni_3_TeO_6_,^14,15^ magnetic chirality is observed, even though the sublattice of magnetic atoms is structurally achiral. There are also materials such as CsCuCl_3_,^16^ MnSi,^17^ and CrNb_3_S_6_,^18^ where the materials are structurally chiral, the magnetic sublattices are also structurally chiral, and magnetic chirality has been observed. Furthermore, even in materials that exhibit both structural and magnetic chirality, the fundamental relation between the two is unclear. Some evidence suggests that in materials that are both structurally and magnetically chiral, the handedness of the magnetic structure matches that of the crystal lattice.^19,20^ However, it has also been reported that if a structurally and magnetically chiral material is doped, the handedness of the chiral magnetic sublattice will flip and become opposite to the material’s structural handedness.^21–24^

The handedness of chiral all-inorganic materials is very difficult to control because in typical crystal growth experiments, an equal amount of left- and right-handed crystals will form as the energies of formation for left- and right-handed materials are equal.^25^ However, incorporating a chiral organic molecule of a single handedness into a material causes the composite organic–inorganic hybrid material to be chiral, maintaining the handedness of the chiral molecule.^26,27^ Until recently, organic–inorganic chiral hybrid magnets were limited to molecular magnets, where chiral ligands are directly bound to the metals,^28–31^ but advances in the synthesis of chiral organic–inorganic hybrid materials, such as perovskites, have allowed new classes of chiral magnetic semiconductors to be synthesized. The first chiral organic–inorganic hybrid halide perovskites were first synthesized in 2003.^27^ In almost all hybrid perovskites, there are no traditional chemical bonds between the organic and inorganic parts of the material; the interactions are electrostatic. In 2016, it was demonstrated that in organic–inorganic hybrid perovskites containing a chiral organic cation, chirality is transferred from the organic to the inorganic lattice despite the lack of chemical bonds between the organic cations and the inorganic framework, and the transfer of chirality was identified by the presence of natural circular dichroism in optical transitions attributed to the material’s inorganic framework.^32^ The mechanism of chirality transfer to the inorganic framework is thought to originate from asymmetric hydrogen bonding interactions inducing chiral distortions in the inorganic framework^33,34^ or from asymmetric orbital polarization induced by the chiral organic cations.^35^

In centrosymmetric materials, the magnetic behavior of a material is determined by the sign of the exchange interaction between electrons on interacting open shell atoms. If only nearest-neighbor magnetic interactions are considered, the classical Heisenberg Hamiltonian can be used: $Ĥ=-J\underset{\langle i,j\rangle }{\sum }{Ŝ}_{i}\cdot {Ŝ}_{j}$ , where *J* is the exchange coupling constant between nearest-neighbor lattice points *i* and *j*, and *Ŝ* is a unit vector that points in the direction of the spin.^36–38^ Under this convention, an interaction is ferromagnetic if *J* is positive and antiferromagnetic if *J* is negative.

In chiral magnets, an antisymmetric contribution to the Hamiltonian must be considered, which leads to an asymmetric electronic structure. For spins ${Ŝ}_{i}$ and ${Ŝ}_{j}$ , ${Ĥ}_{i,j}=\overset{\to }{D_{ij}}\cdot \left({Ŝ}_{i}\times {Ŝ}_{j}\right)$ . This is also called the Dzyaloshinskii–Moriya (DM) interaction.^39,40^ The DM interaction in chiral materials can lead to the formation of helical magnetic spin vortices.^41^ Other chiral magnetic structures can also develop, such as a helical chiral magnetic structure, which arises from the coupling between the DM interaction and a ferromagnetic Heisenberg interaction.^19,39^

The first structurally chiral organic–inorganic hybrid magnetic perovskites were synthesized in 2020, which were the ferromagnetic two-dimensional (2D) materials (R)- and (S)-(MPEA)_2_CuCl_4_ (where MPEA is β-methylphenethylamine).^42^ Since then, additional chiral hybrid materials have been reported including the antiferromagnetic (R)-(MPEA)_4_AgFeCl_8_,^43^ antiferromagnetic (R)- and (S)-(MPEA)_2_MnCl_4_(H_2_O),^44^ and the multiferroic (R)- and (S)-(MPEA)_2_CuCl_4_ exhibiting A-type (or easy-axis)^45^ antiferromagnetism and intralayer ferroelectricity.^46^ However, magnetic chirality (i.e., a spin arrangement with no mirror symmetry that does not change handedness under time reversal)^5^ has not been experimentally demonstrated in any organic–inorganic chiral magnetic material.

In this work, we synthesized the new chiral magnetic perovskite (R)- and (S)-(C_4_H_9_FN)_2_CuCl_4_ (where (C_4_H_9_FN)^+^ is (R)- or (S)-3-fluoropyrrolidinium) and demonstrate that its magnetic structure exhibits magnetic chirality even though the inorganic Cu–Cl sublattice is nearly centrosymmetric, as within reasonable tolerances, it exhibits inversion symmetry in both (R)- and (S)-(C_4_H_9_FN)_2_CuCl_4_. In contrast, racemic (C_4_H_9_FN)_2_CuCl_4_, containing an equal amount of (R) and (S) cations, does not exhibit magnetic chirality, which is expected because the racemic variant is centrosymmetric. Structurally, (R)-, (S)-, and racemic (C_4_H_9_FN)_2_CuCl_4_ are highly distorted two-dimensional (2D) Ruddlesden–Popper phase organic–inorganic hybrid perovskites, where the metal-halide (Cu–Cl) 2D layers or slabs are separated by (C_4_H_9_FN)^+^ organic cations. (R)- and (S)-(C_4_H_9_FN)_2_CuCl_4_ are optically active and have mirrored circular dichroism (CD) spectra, as expected for enantiomorphic chiral materials. Magnetically, (R)-, (S)-, and racemic (C_4_H_9_FN)_2_CuCl_4_ are A-type or easy-axis antiferromagnets, where ferromagnetic interactions dominate within each 2D Cu–Cl layer, but adjacent layers order antiferromagnetically. When the magnetic susceptibility is measured with the field applied perpendicular to the inorganic Cu–Cl layer propagation direction, we observe a transition at the Néel temperature *T_N_* = 2.23 K in all three materials, with a corresponding phase transition observed in specific heat capacity measurements. We also perform AC magnetic susceptibility measurements and find no frequency dependence for (R)- and (S)-(C_4_H_9_FN)_2_CuCl_4_ both above and below the Néel temperature, while racemic (C_4_H_9_FN)_2_CuCl_4_ shows a strong frequency dependence below *T_N_*. Most importantly, the presence of the magnetoelectric effect in (R)- and (S)-(C_4_H_9_FN)_2_CuCl_4_ demonstrates the presence of magnetic chirality, while no magnetoelectric signal is observed for racemic (C_4_H_9_FN)_2_CuCl_4_, indicating an absence of magnetic chirality. Our findings demonstrate that chiral organic cations induce magnetic chirality in the inorganic (magnetic) sublattice, enabling the synthesis of enantiopure samples of magnetically chiral crystals where the structural handedness is defined by the chiral organic cation. Magnetically chiral organic–inorganic hybrid materials including (R)- and (S)-(C_4_H_9_FN)_2_CuCl_4_ can thus be considered for new classes of spintronic devices such as high-speed nonvolatile memory^1,2^ and chiral magnetic field sensors,^4^ as well as provide insight into potential relations between the DM interaction, magnetoelectricity, and the chiral-induced spin selectivity (CISS) effect.^47–49^

## 2 Results and Discussion

### 2.1 Structural Characterization

Figures 1 and S1 depict the crystal structures of (R)-, (S)-, and racemic (C_4_H_9_FN)_2_CuCl_4_. These materials are structurally similar to the Pd analogs that we recently reported^50^ and are highly distorted 2D Ruddlesden–Popper phase hybrid perovskites, where layers of corner-sharing CuCl_6_ octahedra are separated from one another by (C_4_H_9_FN)^+^ cations (Figure 1). Within a CuCl_6_ octahedron, there are four short Cu–Cl bonds that are 2.26 Å in (R)-/(S)-(C_4_H_9_FN)_2_CuCl_4_ and 2.30 Å in racemic (C_4_H_9_FN)_2_CuCl_4_, as well as two long (3.6–3.7 Å) Cu–Cl contacts (Figure 1b and 1d). The observed short Cu–Cl bond lengths are in good agreement with the Cu–Cl bond lengths observed in other Cu­(II)-based metal halides such as CsCuCl_3_ and (PMA)_2_CuCl_4_ (where PMA is phenylmethylammonium).^51^ The elongated Cu–Cl bond contact of approximately 3.60–3.70 Å is due to Jahn–Teller distortions, as noted in other hybrid (organic–inorganic) Cu­(II) halides including MA_2_CuCl_4_ (where MA is methylammonium) and (PMA)_2_CuCl_4_.^51,52^ In (R)-/(S)-(C_4_H_9_FN)_2_CuCl_4_, the two Cu–Cl long contacts have a noticeable difference in length (∼0.1 Å), while the two long contacts are equivalent in length in the racemic analog. Moreover, we observe no significant difference in physical density between the chiral and racemic (C_4_H_9_FN)_2_CuCl_4_ variants (Table 1). (R)-/(S)-(C_4_H_9_FN)_2_CuCl_4_ crystallize in the orthorhombic *P*2_1_2_1_2_1_ (#19) space group, which is in higher symmetry than their Pd analogs, which crystallize in the monoclinic *P*2_1_ space group.^50^ The racemic (C_4_H_9_FN)_2_CuCl_4_ grows in the monoclinic *P*2_1_ */c* (#14) space group and is isostructural to its Pd analog. Both (R)-/(S)- and racemic (C_4_H_9_FN)_2_CuCl_4_ crystals are dark green in color. Detailed single-crystal data and structural refinement parameters are shown in Table 1. Importantly, the inorganic Cu–Cl lattice is nearly centrosymmetric in both (R)- and (S)-(C_4_H_9_FN)_2_CuCl_4_. The greatest deviation from true inversion symmetry (0.193 Å and 0.195 Å in the (R)- and (S)-variants, respectively) is less than the radius of the 50% probability thermal ellipsoid at room temperature, which was determined using the ADDSYM EXACT function in PLATON (Supporting Information); the centrosymmetric inorganic sublattice output by PLATON is compared with the experimental structure in Figure S2.^53^

**Figure 1:**
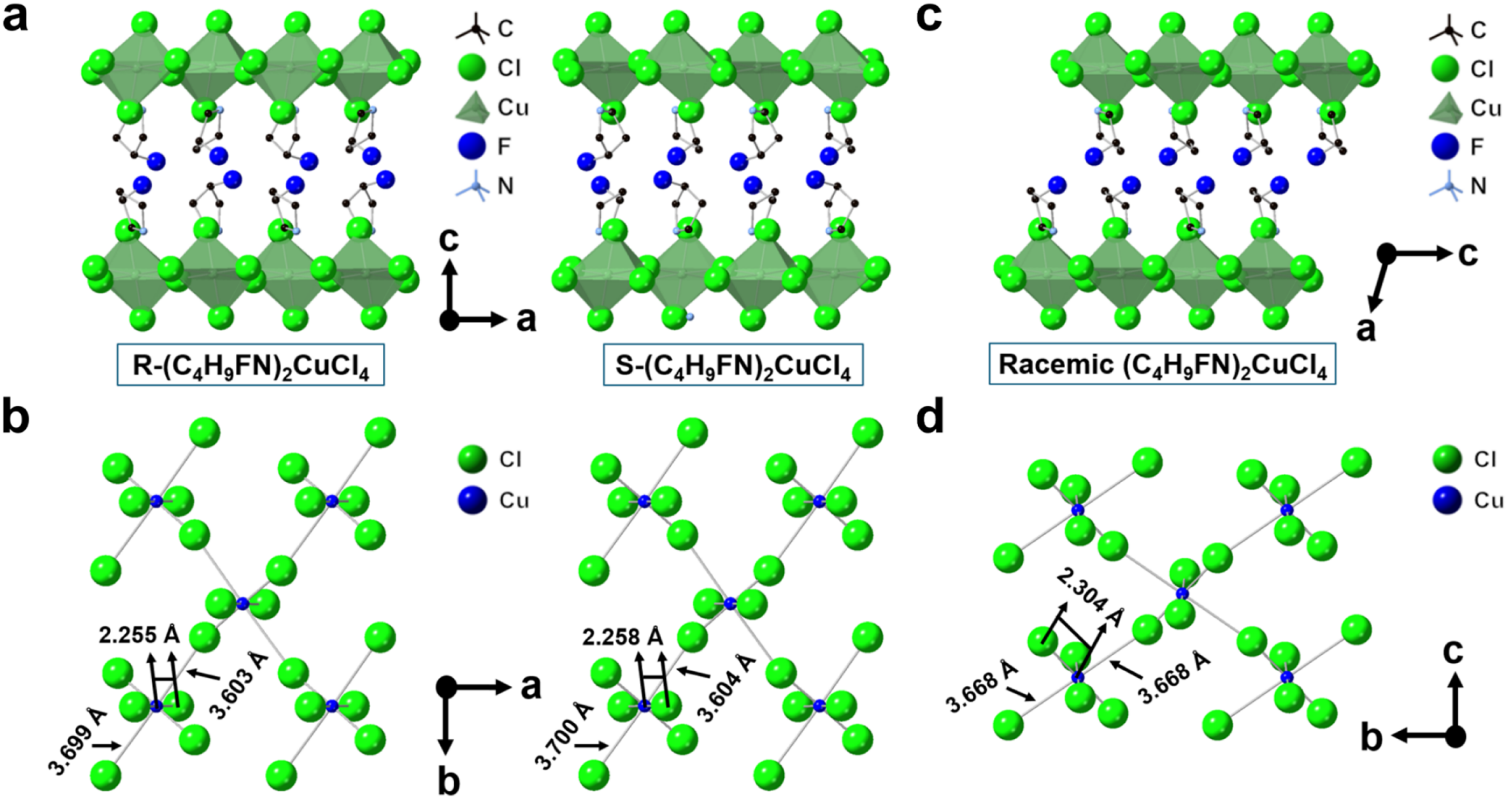
Crystal structures of (a) and (b) (R)- and (S)-(C_4_H_9_FN)_2_CuCl_4_, and (c) and (d) racemic (C_4_H_9_FN)_2_CuCl_4_. Hydrogen atoms are omitted for clarity.

**Table 1:** Crystallographic Collection and Structural Refinement Parameters

Structure	(R)-(C_4_H_9_FN)_2_CuCl_4_	(S)-(C_4_H_9_FN)_2_CuCl_4_	Racemic (C_4_H_9_FN)_2_CuCl_4_
Formula weight (g/mol)	385.58	385.58	385.58
Temperature (K)	300	300	300
Wavelength (Å)	0.71073 (Mo kα)	0.71073 (Mo kα)	1.54178 (Cu kα)
Crystal system	Orthorhombic	Orthorhombic	Monoclinic
Space group	*P*2_1_2_1_2_1_ (#19)	*P*2_1_2_1_2_1_ (#19)	*P*2_1_/*c* (#14)
*Z*	4	4	2
Unit cell parameters	*a* = 7.7559(4) Å	*a* = 7.7598(3) Å	*a* = 10.9445(2) Å
	*b* = 8.7973(4) Å	*b* = 8.8006(3) Å	*b* = 9.0521(2) Å
	*c* = 21.1758(10) Å	*c* = 21.1880(6) Å	*c* = 7.56760(10) Å
			β = 105.4050(10)°
Volume (Å^3^)	1444.85(12)	1446.95(9)	722.79(2)
Density (g/cm^3^)	1.773	1.770	1.772
Absorption coefficient (μ) (mm-1)	2.253	2.250	8.994
θ_min_ *- θ*_max_ (°)	5.014 to 61.102	5.012 to 61.218	12.886 to 144.562
Reflections collected	27540	29572	16638
Independent reflections	4428	4446	1428
*Ra* indices (*I* > 2σ(*I*))	*R*_1_ = 0.0402	*R*_1_ = 0.0367	*R*_1_ = 0.0214
	*wR*_2_ = 0.0842	*wR*_2_ = 0.0903	*wR*_2_ = 0.0604
Goodness-of-fit on *F*2	1.048	1.017	1.063
Largest diff. peak/hole (e-/Å3)	0.63/–0.60	0.53/–0.56	0.31/–0.26
Flack parameter	0.009(15)	0.027(11)	-

Figure S3 shows the measured experimental powder X-ray diffraction (PXRD) pattern of (C_4_H_9_FN)_2_CuCl_4_ compared to the calculated pattern. The experimental PXRD pattern shows good agreement with the calculated pattern given by single-crystal XRD measurements, indicating the sample that we used for PXRD analysis is phase-pure. To check the stability of (C_4_H_9_FN)_2_CuCl_4_ against ambient air exposure, we performed periodic PXRD measurements over time to monitor changes in the PXRD pattern (see Figure S4). Based on the obtained PXRD data, both (R)- and (S)-(C_4_H_9_FN)_2_CuCl_4_ show a decrease in peak intensity after 4 weeks of exposure to air, suggesting reduced crystallinity but no transition to a new crystalline phase. Moreover, after 4 weeks, we observed a color change on the powder sample’s surface, implying (C_4_H_9_FN)_2_CuCl_4_ is not completely stable in air, though the degradation occurs over a time frame of weeks to months. Degradation of single crystals is likely insignificant compared to powder due to the much smaller surface area-to-volume ratio.

### 2.2 Optical Characterization

Figure 2 shows the obtained ultraviolet–visible (UV–vis) absorbance and circular dichroism (CD) data using the deposited chiral and racemic (C_4_H_9_FN)_2_CuCl_4_ films. X-ray diffraction patterns of the (R)-/(S)-(C_4_H_9_FN)_2_CuCl_4_ and racemic (C_4_H_9_FN)_2_CuCl_4_ thin films are shown in Figure S5. CD spectra are corrected for linear dichroism and linear birefringence effects (Figure S6).^54–56^ The UV–vis absorbance data show two distinct absorption features: a peak centered at 380 nm and a shoulder at ∼450 nm. Based on the CD spectra, (R)- and (S)-(C_4_H_9_FN)_2_CuCl_4_ thin films are optically active, indicating that the organic cation transfers chirality into the inorganic matrix. As expected, the CD spectra for (R)- and (S)-(C_4_H_9_FN)_2_CuCl_4_ exhibit features of opposite signs, with maxima/minima at 383 and 353 nm with the spectra intersecting at 367 nm. The CD sign inversion at 367 nm is an indication of the Cotton effect, which is expected to be observed near a feature in the absorption spectrum.^56^ As expected, a negligible CD signal was observed for racemic (C_4_H_9_FN)_2_CuCl_4_. This observation is similar to the Pd analog that we reported,^50^ where (R)-/(S)-(C_4_H_9_FN)_2_PdCl_4_ are optically active, while we observe no CD signal from (R)-/(S)-(C_5_H_12_N)_2_PdCl_4_ ((C_5_H_12_N)^+^ is 3-methylpyrrolidinium), highlighting the importance of the chiral organic cation and the interaction between the chiral cation and the inorganic framework in determining the chiroptical properties of the material. Furthermore, the calculated absolute values of the anisotropy factor $g_{CD}=\frac{CD\left(mdeg\right)}{32980\times A}$ , where A is the absorbance, for (R)- and (S)-(C_4_H_9_FN)_2_CuCl_4_ at 415 nm are 0.63 × 10^–4^ and 1.44 × 10^–4^ (Figure S7), respectively. These values are similar to those of (R)-/(S)-(THBTD)_2_CuCl_6_ (2.8 × 10^–4^ for (R), 6.8 × 10^–4^ for (S), where THBTD is 4,5,6,7-tetrahydro-benzothiazole-2,6-diamine), and an order of magnitude lower than those of (R)-/(S)-(1,2-NEA)_2_CuCl_4_ (∼2.9 × 10^–3^, where 1,2-NEA is 1-(2-naphthyl)­ethylammonium).^57,58^

**Figure 2:**
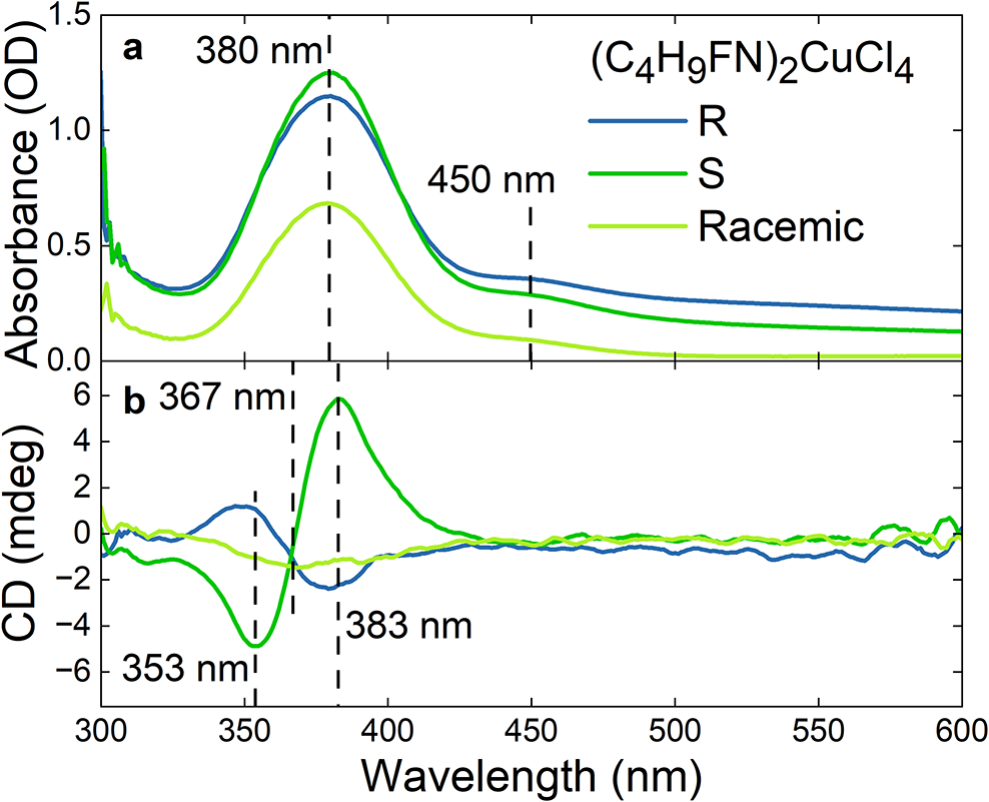
(a) UV–vis absorption and (b) CD spectra of (R)-, (S)-, and racemic (C_4_H_9_FN)_2_CuCl_4_ thin films.

### 2.3 Magnetic Characterization

Figure 3 shows the zero-field-cooled inverse magnetic susceptibility vs temperature for powdered (R)-, (S)-, and racemic (C_4_H_9_FN)_2_CuCl_4_ at an applied field of 1000 Oe. At temperatures far from any phase transitions, the inverse susceptibility 1/χ should be linear with temperature following the Curie–Weiss law $\frac{1}{\left(\chi -\chi_{0}\right)}=\left(\frac{1}{C}\right)T-\left(\frac{\theta_{CW}}{C}\right)$ , where χ is the susceptibility, χ_0_ is the temperature-independent contribution to the susceptibility that likely comes from core diamagnetism,^59^ *C* is the Curie constant, and *θ_CW_* is the Weiss temperature; the x-intercept of the extrapolated linear fit is *θ_CW_*. This relation holds true for our data: the inverse susceptibility from 50–300 K is well-fit by the Curie–Weiss law (all fits have adjusted R^2^ values greater than 0.99), and the fits are shown as solid lines in Figure 3 with dashed lines extrapolating the linear fit to *θ_CW_*, and fit parameters are listed in Table 2. The effective moment was calculated using the equation $\mu_{eff}=\sqrt{\frac{3k_{B}}{N_{A}\mu_{B}^{2}}C}$ , expressed in Bohr magnetons (*μ*_*B*_). The positive Weiss temperatures *θ_CW_*, which can be observed in Figure 3 by the positive value of the *x*-intercept, indicate that ferromagnetic interactions dominate. For all variants, the effective moment is greater than the spin-only moment for an *S* = 1/2 system (*μ* = 1.73*μ*_B_), which is commonly observed in Cu­(II)-containing materials and results from spin–orbit coupling interactions.^60,61^ Figure S8 shows both zero-field-cooled and field-cooled susceptibility measurements on the chiral and racemic variants. These traces overlap perfectly, making the presence of bulk ferromagnetism unlikely despite the positive Weiss temperatures.^62^

**Figure 3:**
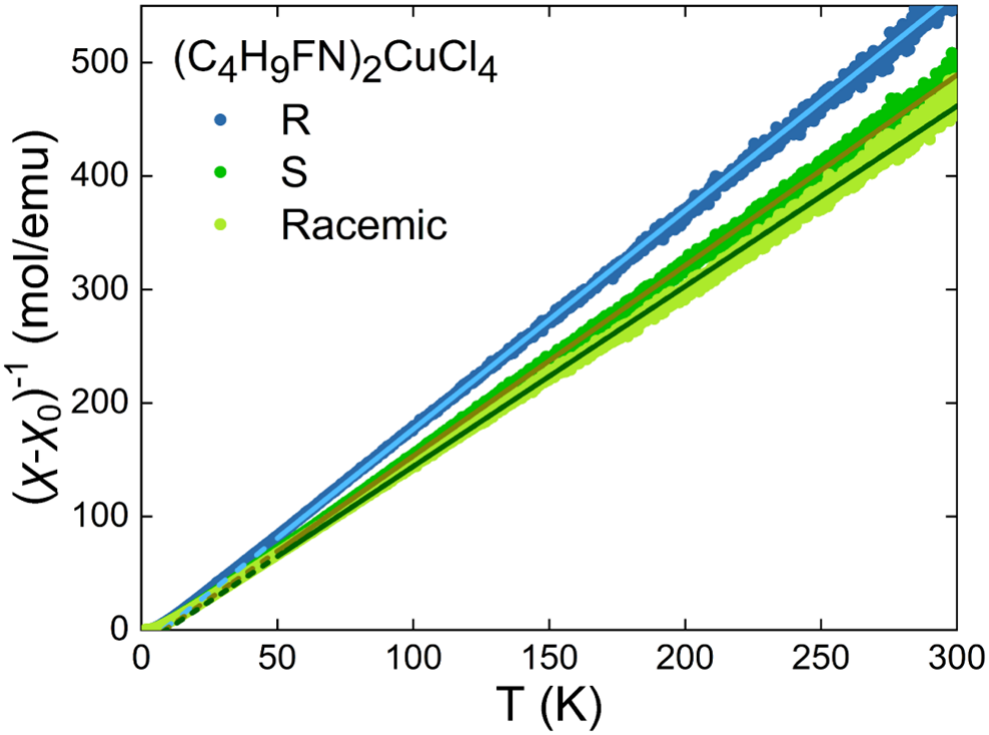
Plot of the inverse susceptibility (*χ* – *χ*_0_)^−1^ versus temperature for powder samples of (R)-, (S)-, and racemic (C_4_H_9_FN)_2_CuCl_4_. Curie–Weiss fits are shown as solid lines with extrapolations of the fits as dashed lines.

**Table 2:** Parameters Obtained from Curie–Weiss Fits of the Magnetic Susceptibility Data for (C_4_H_9_FN)_2_CuCl_4_ Powder

	(R)	(S)	Racemic
μ_eff_ (μ_B_)	2.04	2.18	2.24
θ_CW_ (K)	8.07	8.69	9.23
χ_0_ (emu/mol)	–0.00131	–0.00109	–0.00164

We further carried out magnetic characterizations for the synthesized chiral and racemic (C_4_H_9_FN)_2_CuCl_4_ single crystals; field-cooled measurements are shown in Figure 4. The measured magnetic susceptibility data vs temperature (at an applied field of 100 Oe) shown in Figure 4a-4d show that both chiral and racemic (C_4_H_9_FN)_2_CuCl_4_ single crystals behave as A-type antiferromagnets,^63^ where ferromagnetic behavior dominates when measured along the inorganic Cu–Cl layer propagation direction (*ab* plane for (R)-/(S)-(C_4_H_9_FN)_2_CuCl_4_, *bc* plane for racemic (C_4_H_9_FN)_2_CuCl_4_, see Figure 1), though we do not observe a ferromagnetic phase transition. As field-cooled measurements are plotted, a downturn in the susceptibility below the critical temperature would not be present for ferromagnets, supporting our hypothesis that the chiral and racemic variants are all A-type antiferromagnets. Our findings are consistent with the magnetic behavior of previously reported achiral Cu­(II) 2D Ruddlesden–Popper phase (*i.e.*, Cu­(II) ions in adjacent layers are staggered) perovskites.^64–66^ A wide variety of magnetic behaviors have been reported in organic–inorganic hybrid 2D Cu­(II) perovskites, where the magnetic behavior depends on the relative strengths of inter- and intralayer coupling as well as whether the Cu­(II) ions in adjacent layers are staggered or eclipsed; the intralayer ferromagnetic coupling decreases as the length of the long Cu-X contact increases,^67,68^ and the interlayer antiferromagnetic coupling decreases as the distance between Cu-X layers increases.^69^ When measured perpendicular to the Cu–Cl layer propagation direction, an antiferromagnetic phase transition at a Néel temperature *T*_*N*_ = 2.23 K is observed for both chiral and racemic (C_4_H_9_FN)_2_CuCl_4_. This transition is also observed in the heat capacity data (Figures 4e–f and S9), which supports the existence of a magnetic phase transition for chiral and racemic (C_4_H_9_FN)_2_CuCl_4_ at 2.23 K. To determine the magnetic entropy, we fit the heat capacity data from 20 to 300 K using the double Debye model (Figure S9) to account for the distribution of phonon energies in both the organic and inorganic moieties.^70,71^ The fitting yields two Debye temperatures: 335.4 and 120.3 K (for chiral (C_4_H_9_FN)_2_CuCl_4_), and 383.2 and 130.2 K (for racemic (C_4_H_9_FN)_2_CuCl_4_), respectively. After subtracting the extrapolated double Debye model fit from the total heat capacity, we extract a magnetic entropy of ∼5.74 J·mol^–1^·K^–1^ and ∼4.94 J·mol^–1^·K^–1^, respectively, for chiral and racemic (C_4_H_9_FN)_2_CuCl_4_, both of which are close to the expected value (*R* ln(2) = 5.76 J·mol^–1^·K^–1^) for magnetic order in an S = 1/2 material. When the magnetization is measured parallel to the Cu–Cl layer propagation direction, no antiferromagnetic phase transition is observed, with only a small downturn in the χT plot below *T*_*N*_ observed for the both the chiral and racemic variants. The presence of stronger ferromagnetic interactions in the single-crystal susceptibility measurements is consistent with the greater Weiss temperature of the racemic variant (Table 2).

**Figure 4:**
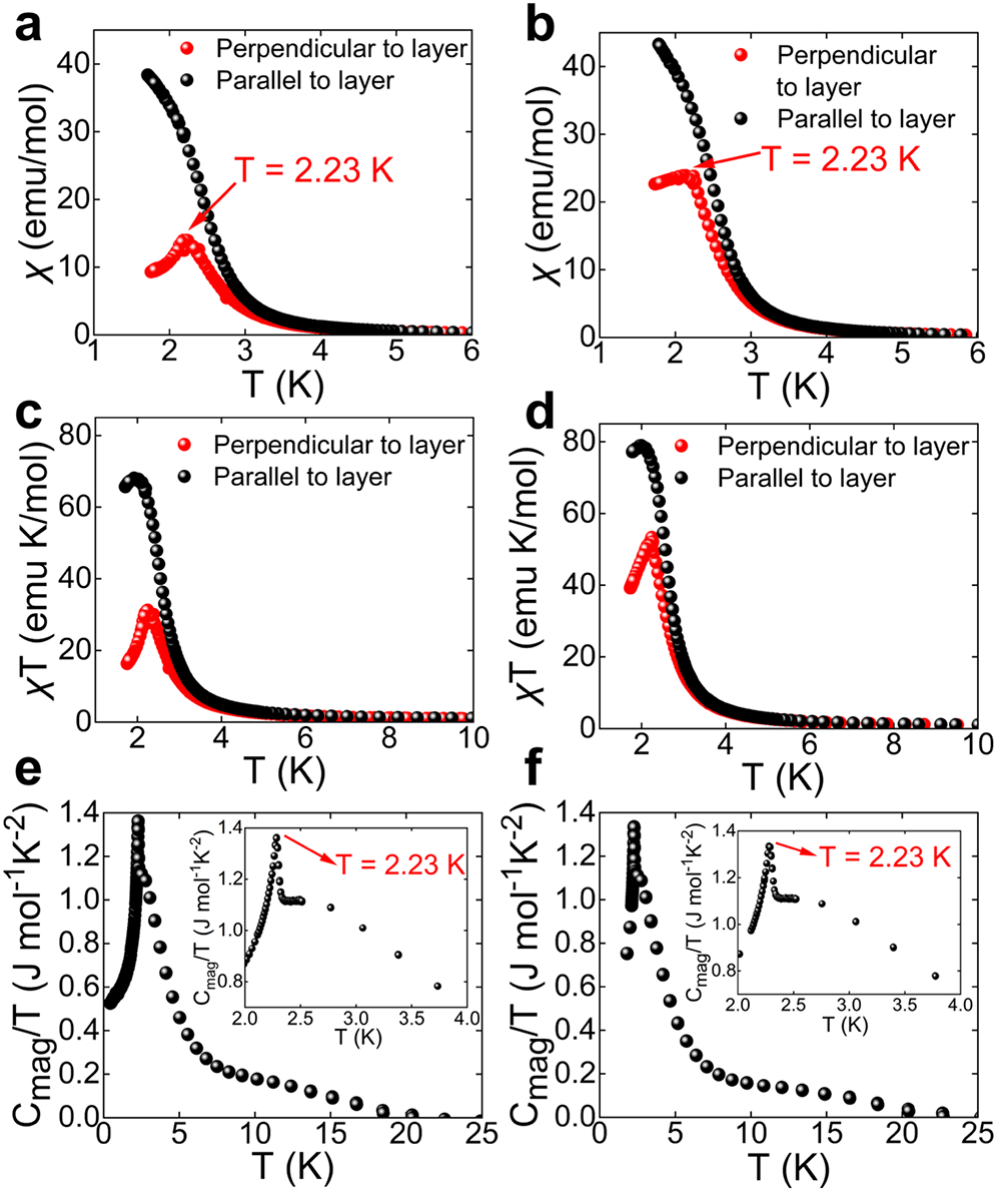
Plots of the magnetic susceptibility and magnetic specific heat capacity data for (a), (c),(e) chiral (C_4_H_9_FN)_2_CuCl_4_ and (b), (d),(f) racemic (C_4_H_9_FN)_2_CuCl_4_ single crystals.

Field-dependent magnetization measurements at 1.8 K are shown in Figure 5. Based on the measured data, as expected, both chiral and racemic (C_4_H_9_FN)_2_CuCl_4_ exhibit para- or ferromagnetic behavior, consistent with our hypothesis that these materials are A-type (easy-axis) antiferromagnets, as hysteresis is present. However, unlike the racemic sample, there is a hesitation in the change of magnetic moment around the zero magnetic field region for chiral (C_4_H_9_FN)_2_CuCl_4_ (Figure 5a). This “hesitation” in the change of magnetic moment is likely a spin-flip transition, where once a critical field is reached, the spins rapidly rearrange to align with one another, and the moment then quickly saturates.^45,72,73^ A similar spin-flip transition has has been observed in EA_2_CuCl_4_ (EA = ethylammonium)^45^ We note that this is distinct from a spin-flop transition, in which the transition is second order, where the moment slowly changes with increasing field after the sudden change in magnetization.^45,66,68^ Spin-flop transitions are much more common than spin-flip transitions in 2D hybrid organic–inorganic magnetic metal halide materials because of the antiferromagnetic coupling, and examples of materials that exhibit spin-flop transitions are EA_2_CuBr_4_, (PEA)_2_MnCl_4_, and (PEA)_2_CuCl_4_ (where PEA = 2-phenethylammonium);^74–77^ EA_2_CuCl_4_ exhibits both a spin-flip and spin-flop transition, depending on temperature and field.^45^ In addition, spin-flip transitions are indicative of greater magnetocrystalline anisotropy than spin-flop transitions,^72,78^ indicating that (R)- and (S)-(C_4_H_9_FN)_2_CuCl_4_ exhibit larger magnetocrystalline anisotropy than most other reported hybrid magnetic perovskites.

**Figure 5:**
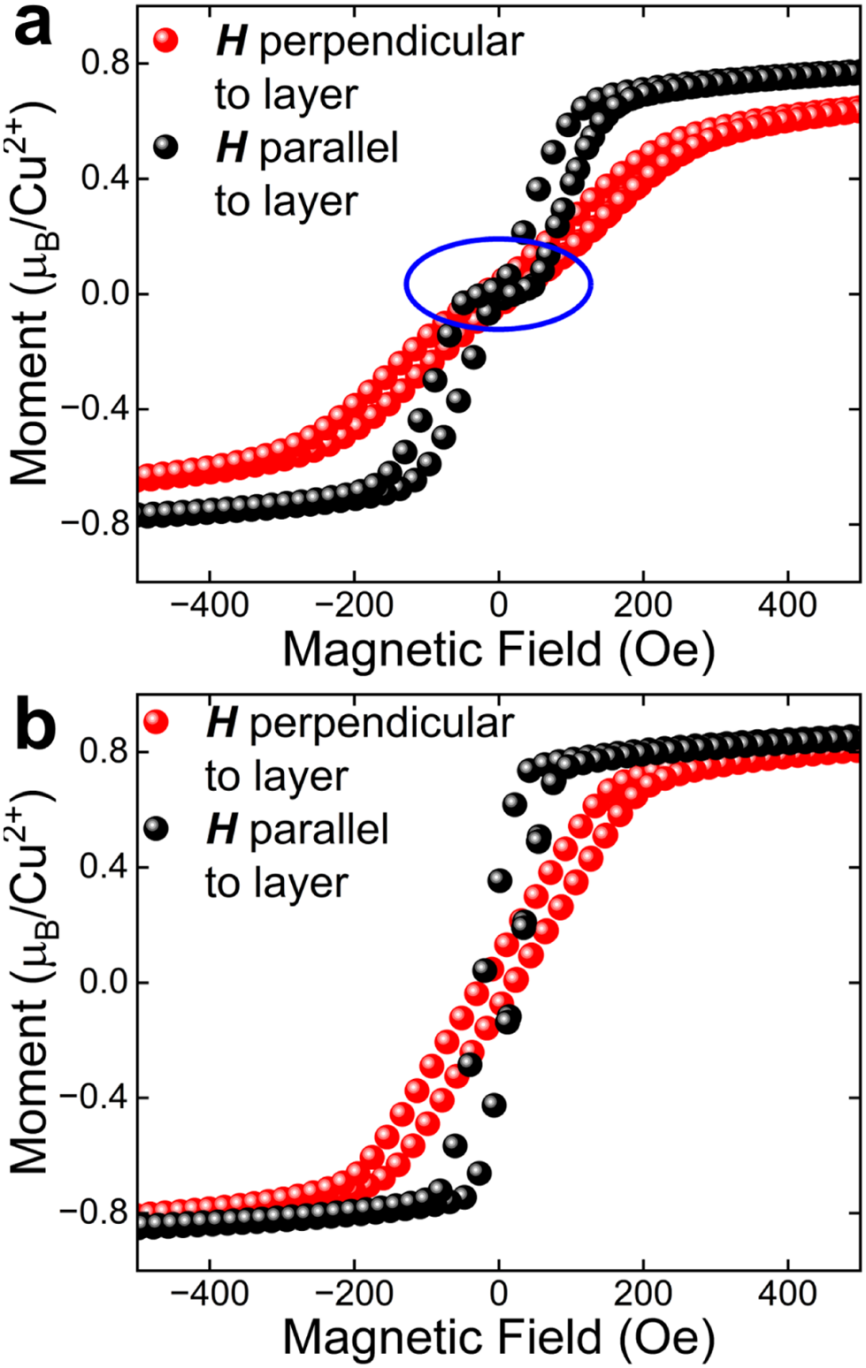
Plots of the magnetic moment vs magnetic field H (at *T* = 1.8 K) for (a) chiral and (b) racemic (C_4_H_9_FN)_2_CuCl_4_ single crystals. The blue circle in panel (a) is likely a spin-flip transition.

In addition to measuring the magnetic moment, we carried out frequency-dependent AC magnetization measurements for the chiral and racemic samples (Figures 6 and S10). Figure 6a and 6b present the temperature-dependent in-phase component (*χ’*) of the AC magnetic susceptibility data for (R)- and racemic (C_4_H_9_FN)_2_CuCl_4_ powder, respectively, measured under a 1.5 Oe AC magnetic field. We once more observed the Néel temperature transition at 2.23 K in both the chiral and racemic materials. For chiral (C_4_H_9_FN)_2_CuCl_4_, the measured AC susceptibility data show no frequency dependence. However, for the racemic material, the moment decreases with increasing frequency below the Néel temperature (Figure 6b). Despite the frequency dependence of the susceptibility, the racemic variant does not form a spin glass because the Néel temperature is independent of frequency.^79,80^ We hypothesize that the difference in frequency dependence between the chiral and racemic materials originates from the much higher strength of the antiferromagnetic interaction in the chiral material compared to the racemic, which can be observed in both the temperature- and field-dependent magnetic data (Figures 4 and 5), especially by the presence of the spin-flip transition in the chiral variant. At low field, antiferromagnetic behavior dominates in the chiral material regardless of the direction of the applied field, so the frequency-dependent hysteresis that would be expected for a ferromagnet is not observed. In contrast, the racemic variant has much weaker antiferromagnetic interactions despite having an identical Néel temperature, so hysteresis can be seen in the frequency dependence of the AC susceptibility, indicative of the presence of domain walls that are present in ferro- and ferrimagnets.^81^ The behavior of the out-of-phase component of the AC susceptibility (*χ’’*) is also consistent with this explanation—it is approximately 0 for (R)- and (S)-(C_4_H_9_FN)_2_CuCl_4_ (Figures 6c and S10), indicative of antiferromagnetism, but shows a dramatic frequency dependence below ∼2.3 K in racemic (C_4_H_9_FN)_2_CuCl_4_ (Figure 6d), indicative of domain-wall ferro- or ferrimagnetism. To conclusively demonstrate the presence of long-range magnetic order, neutron diffraction measurements must be performed.

**Figure 6:**
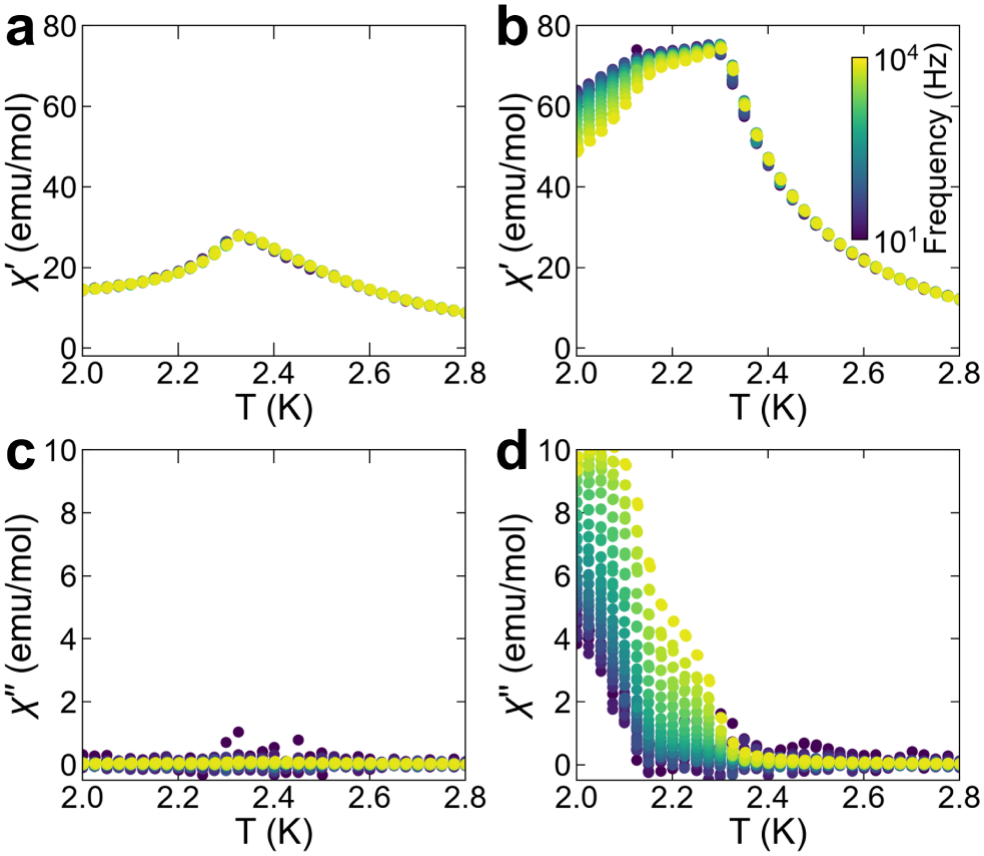
In-phase (*χ’*) component of AC susceptibility for (a) (R)-(C_4_H_9_FN)_2_CuCl_4_ and (b) racemic (C_4_H_9_FN)_2_CuCl_4_. Out-of-phase (*χ’’*) component of AC susceptibility for (c) (R)-(C_4_H_9_FN)_2_CuCl_4_ and (d) racemic (C_4_H_9_FN)_2_CuCl_4_.

Figure S11 presents the resistivity data for (C_4_H_9_FN)_2_CuCl_4_ samples. The crystal shows a resistivity of ∼10^4^ Ω·cm (measured along the Cu–Cl layer propagation direction), while, when measured perpendicular to the layer propagation direction, a resistivity of ∼10^7^ Ω·cm is obtained. The higher resistivity is consistent with the presence of organic cations between inorganic layers.

Magnetoelectric data are shown in Figure 7. Importantly, the chiral variant shows a magnetoelectric signal, demonstrating the existence of chiral magnetic order in the material. The chiral crystal structure (space group *P*2_1_2_1_2_1_, no. 19) breaks spatial inversion symmetry and permits higher-order coupling between the lattice and magnetization. Specifically, when an in-plane magnetic field containing both *a*- and *b*-axis components is applied, the *a*-axis field, together with the structural chirality, effectively induces a toroidal moment along the *a*-axis. This induced toroidal moment, in combination with the magnetic field component along *b*, further generates an electric polarization along the *c*-axis, i.e., a second-order magnetoelectric effect.^5^ Notably, this mechanism does not require long-range chiral magnetic order from a symmetry perspective (short-range chiral correlations are sufficient), but lower temperatures are beneficial as they suppress thermal fluctuations and enhance the magnetization response. As shown in Figure 7, the chiral variant shows a clear magnetoelectric signal at 2 K, with the current reversing its sign upon reversing the magnetic field, consistent with the presence of a toroidal moment and the chirality-induced second-order magnetoelectric mechanism described above. In contrast, the racemic sample shows no detectable magnetoelectric response when an in-plane magnetic field (here, containing both *b*- and *c*-axis components) is applied, indicating the absence of a field-induced toroidal moment, as expected for an achiral material.

**Figure 7:**
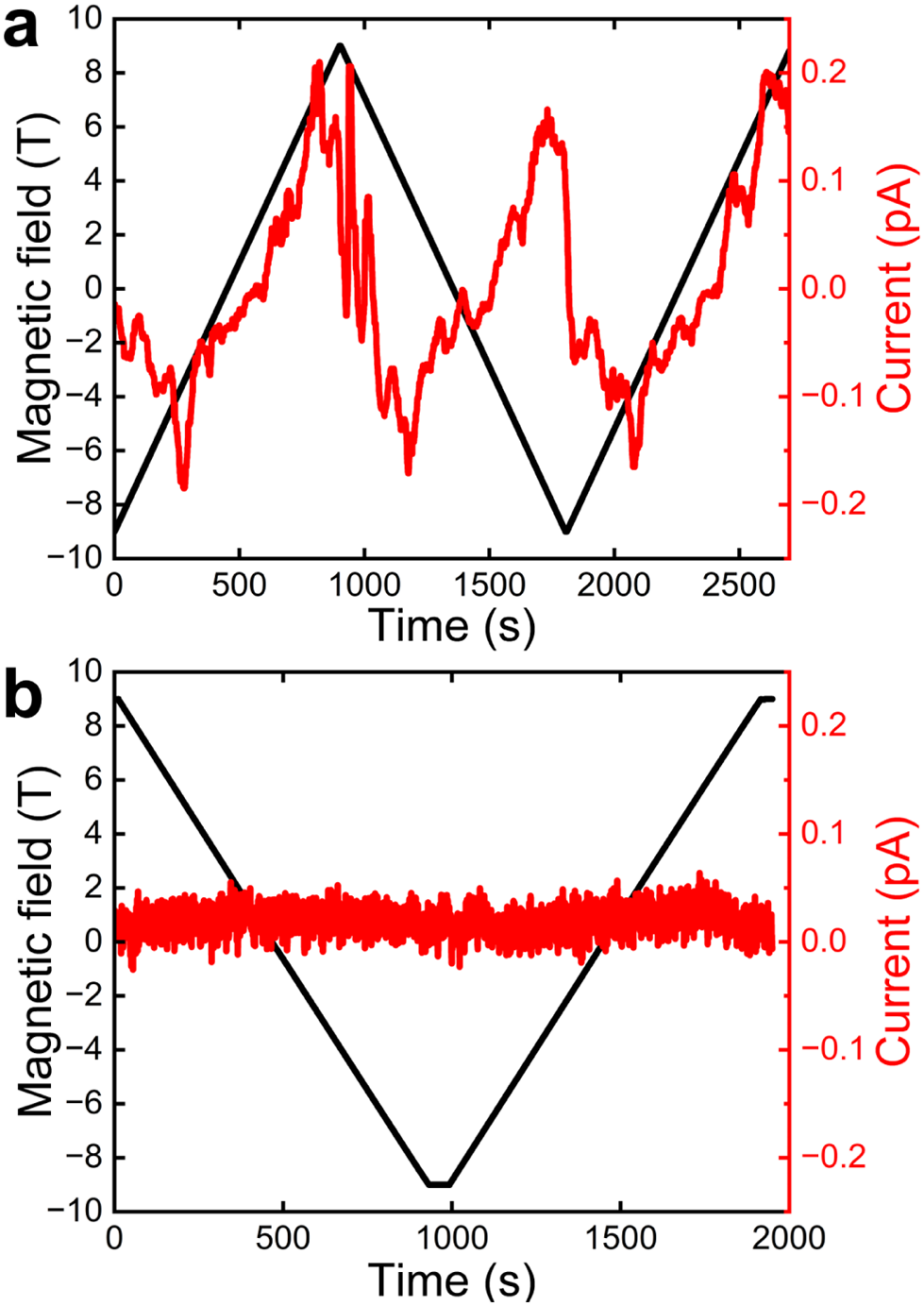
Magnetoelectric data collected at 2 K on (a) chiral and (b) racemic (C_4_H_9_FN)_2_CuCl_4_ crystals. The magnetic field is applied along the in-plane direction, and the out-of-plane magnetoelectric current is collected.

## 3 Conclusion

To summarize, we demonstrate that in the chiral organic–inorganic hybrid perovskite (C_4_H_9_FN)_2_CuCl_4_, the chiral organic cations induce the formation of chiral magnetic order in the nearly centrosymmetric magnetic sublattice. For both chiral and racemic (C_4_H_9_FN)_2_CuCl_4_, a magnetic phase transition at *T*_*N*_ = 2.23 K is observed when measured perpendicular to the Cu–Cl layer propagation direction, and specific heat capacity measurements reveal a magnetic entropy consistent with S = 1/2 magnetic order for both chiral and racemic (C_4_H_9_FN)_2_CuCl_4_. The strength of the interlayer antiferromagnetic interactions is stronger in the chiral variants than in the racemic variant. Finally, the presence of the magnetoelectric effect in chiral (C_4_H_9_FN)_2_CuCl_4_ supports the presence of magnetic chirality in (R)- and (S)-(C_4_H_9_FN)_2_CuCl_4_, while the lack of a magnetoelectric effect in racemic (C_4_H_9_FN)_2_CuCl_4_ emphasizes that the structural chirality of (R)- and (S)-(C_4_H_9_FN)_2_CuCl_4_ is responsible for the toroidal magnetic signal. These findings demonstrate the importance of the chiral organic cations in the magnetic behavior of organic–inorganic hybrid materials, warranting additional study to determine the magnetic crystal structures of these and other chiral magnetic hybrid materials and their potential application in magnetic information processing and spin–orbit torque memory devices. Furthermore, the combination of magnetic chirality and large magnetocrystalline anisotropy suggests that the properties of (R)- and (S)-(C_4_H_9_FN)_2_CuCl_4_ may provide insight into the origin of the CISS effect and its relation to asymmetric Dzyaloshinskii–Moriya interactions that are responsible for chiral magnetism.^82^

## Supplementary Material



## Notes

Experimental section, additional depictions of crystal structures, powder X-ray diffraction (PXRD) patterns, linear dichroism and linear birefringence correction to circular dichroism spectra, plot of the anisotropy factor (g_CD_), additional susceptibility data on powder samples, additional heat capacity data, additional AC susceptibility data, electronic measurement results, output of PLATON ADDSYM (PDF)
