# Supplementary material for: Chirality Transfer to the Magnetic Sublattice in the Hybrid Perovskite (R)-/(S)-3-Fluoropyrrolidinium Copper(II) Chloride
Source: J Am Chem Soc. 2026 Jul 31;148(31):33895–904. doi: 10.1021/jacs.6c11636 (PMC13474938; doi:10.1021/jacs.6c11636)
Supplement: Supplementary file 1 [file ja6c11636_si_001.pdf]

Supporting Information

Chirality Transfer to the Magnetic Sublattice in the Hybrid Perovskite (R)-/(S)-3-Fluoropyrrolidinium Copper(II) Chloride

Zheng Zhang[a], Mingyu Xu[b], Jose L. Gonzalez Jimenez[b], Stephen Zhang[c], Weiwei Xie[b], Xianghan Xu[d], and Daniel B. Straus[a*]

[a]Department of Chemistry, Tulane University, New Orleans, LA, USA 70118
[b]Department of Chemistry, Michigan State University, East Lansing, MI, USA 48824
[c]Department of Chemistry, Princeton University, Princeton, NJ, USA 08540
[d]School of Physics and Astronomy, University of Minnesota, Twin Cities, MN, USA 55455
(*Author to whom correspondence should be addressed: dstraus@tulane.edu)

1. Experimental Section

Materials

Copper (II) chloride (anhydrous, 99% extra pure) was purchased from Acros Organics. (R)-3-fluoropyrrolidine hydrochloride and (S)-3-fluoropyrrolidine hydrochloride were purchased from Ossila and Ambeed, respectively. Hydrochloric acid (36.5-38%) was purchased from VWR International. Dimethylformamide (DMF, ≥99.8%, purity grade: spectranalyzed) was purchased from Fisher Scientific.

Crystal Growth

To synthesize (R)- or (S)-$(C_4H_9FN)_2CuCl_4$ crystals, 2 mmol (R)-3-fluoropyrrolidine hydrochloride or 2 mmol (S)-3-fluoropyrrolidine hydrochloride was first dissolved in 3 mL HCl acid. Then, 2 mmol $CuCl_2$ was added to the solution. The solution was then vigorously stirred until a

clear solution was obtained. For the synthesis of racemic $(C_4H_9FN)_2CuCl_4$ crystals, 1 mmol (R)-3-fluoropyrrolidine hydrochloride and 1 mmol (S)-3-fluoropyrrolidine hydrochloride were added to 3 mL HCl acid. 2 mmol $CuCl_2$ was then added, and a clear solution was obtained after stirring. Crystal growth experiments were performed by the evaporation of solvent from the prepared solutions in open air. Typically, mm-to-cm crystals scale are obtained after one week.

X-Ray Diffraction (XRD) Measurements

Single crystal X-ray diffraction data were collected at room temperature using a Bruker D8 Quest diffractometer equipped with a Photon III 7 detector using Mo Kα radiation or a Bruker D8 Venture diffractometer equipped with a Photon III 14 detector using Cu Kα X-ray irradiation. The crystal structures were solved using ShelXT[1] and refined using ShelXL[2] within the Olex2 GUI.[3]

For powder X-ray diffraction measurements, (R)-, (S)-, or racemic $(C_4H_9FN)_2CuCl_4$ crystals were ground into a powder using a mortar and pestle, which was measured using a Malvern Panalytical Aeris diffractometer equipped with a PIXcel3D detector using Cu Kα radiation. To check stability against ambient air exposure, $(C_4H_9FN)_2CuCl_4$ powders were stored under ambient conditions in the dark, and periodic PXRD measurements were taken to monitor the PXRD pattern change.

Thin Film Preparation

(R)-, (S)-, and racemic $(C_4H_9FN)_2CuCl_4$ thin films were spin-cast onto 25x25x1mm glass substrates. Substrates were cleaned by successive sonication in the following solvents or solutions: 5% v/v aqueous solution of Hellmanex III detergent in deionized water; deionized water (3x); 1 M hydrochloric acid; deionized water (3x); and ethanol (3x). The substrates were then treated in a UV-Ozone cleaner located in a nitrogen glove box for 30 min.

To prepare the films, 30 mg of (R)-, (S)-, or racemic $(C_4H_9FN)_2CuCl_4$ crystals were dissolved in 0.4 mL methanol. The solutions were spin-cast onto the cleaned glass substrates at 1000 RPM for 45 s in a nitrogen glove box.

UV-Vis Absorbance and Circular Dichroism (CD) Measurements

UV-Vis absorbance data were collected on thin films of (R)-, (S)-, and racemic $(C_4H_9FN)_2CuCl_4$ using an Agilent Cary 50 Bio UV-Vis spectrometer in transmission. CD spectra were collected using an OLIS DSM 1000 CD spectrometer in transmission on the same thin films used in absorbance measurements. The CD spectrometer was calibrated using *d*-camphorsulfonic acid as a standard.[4] To account for linear dichroism (LD) and linear birefringence (LB) effects, the CD spectrum for each sample was measured twice (Figure S6), once with the film facing towards the detector and once with the film facing away from the detector. Averaging the two spectra gives the CD spectrum corrected for LD and LB effects.[5–7]

Electrical Characterization

For current-voltage (I-V) and space-charge-limited-current (SCLC) measurements, conductive silver paste (purchased from Ted Pella, Inc., PELCO conductive silver paint) was used to attach copper wires to the top and bottom of a (R)-$(C_4H_9FN)_2CuCl_4$ crystal (vertical-type device structure). The crystal was then placed in an in-house built sample measurement box, which is connected to a Keithley 2636A sourcemeter. The I-V and SCLC testing were performed at room temperature and in a dark environment. For photo-response measurement, crystal was exposed to 365 nm light illumination (wattage 5 watts, bulb voltage 5 volts). The light was turned on and off to measure the crystal's response to light exposure.

Magnetic Characterization

A Quantum Design Dynacool PPMS equipped with the ACMS II magnetization accessory was used to measure the temperature- and field-dependent DC magnetization of single crystal and powder samples of (R)-, (S)-, and racemic $(C_4H_9FN)_2CuCl_4$ as well as AC magnetization on powder samples. The temperature-dependent magnetization was measured at an applied field of 100 Oe on single crystal samples and 1000 Oe on powder samples. The susceptibility χ is defined as M/H, where M is the magnetization and H the applied field.

Heat Capacity Measurements

Heat capacity measurements between 1.8 and 300 K were carried out using the Quantum Design Dynacool PPMS Heat Capacity accessory. Heat capacity measurements below 1.8 K used a Quantum Design ^{3}He dilution refrigerator insert for the PPMS.

Magnetoelectricity Measurements

Chiral and racemic crystals were cleaved into plate-like samples with a thickness of 0.5(1) mm. Silver paste was applied to form top and bottom electrodes. Due to thermal decomposition of the material, the electrodes were cured at room temperature over an extended period. The electrode areas for both devices are 2.0(5) mm^2. The devices were then cooled to 2 K in a PPMS under a 9 T in-plane magnetic field applied along (110) for chiral crystals and (011) for racemic crystals, and an applied out-of-plane electric field of 2 kV/cm. Magnetoelectric current signals at 2 K were measured using a Keithley 617 electrometer while sweeping the in-plane magnetic field at a rate of 200 Oe/s.

2. Additional Depictions of Crystal Structures

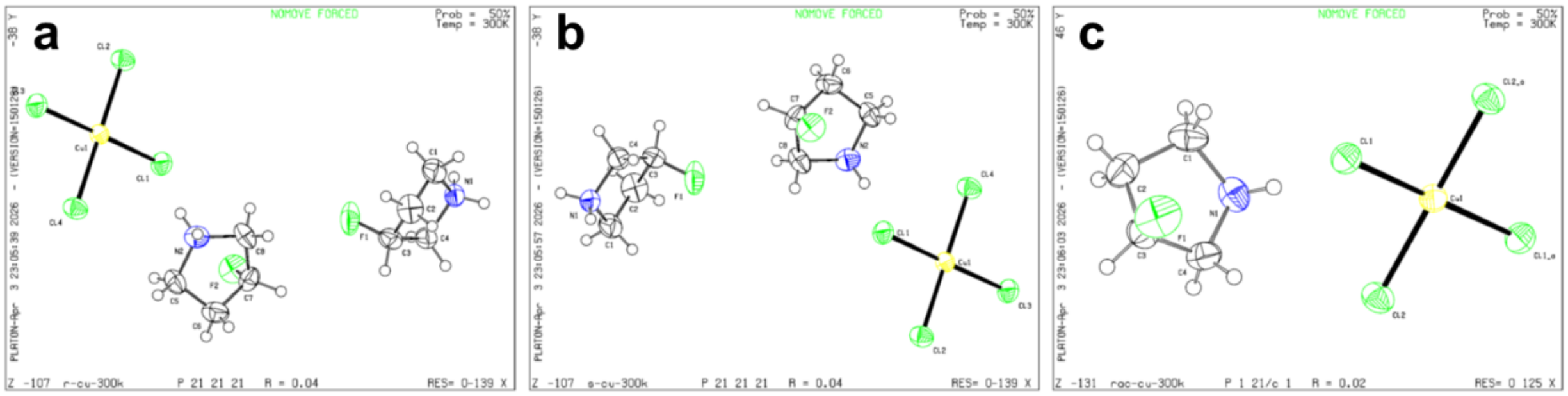

Figure S1. Asymmetric units of **(a)** (R)-, **(b)** (S)-, and **(c)** racemic $(C_4H_9FN)_2CuCl_4$ crystal structures, with atoms represented as 50% probability thermal ellipsoids.

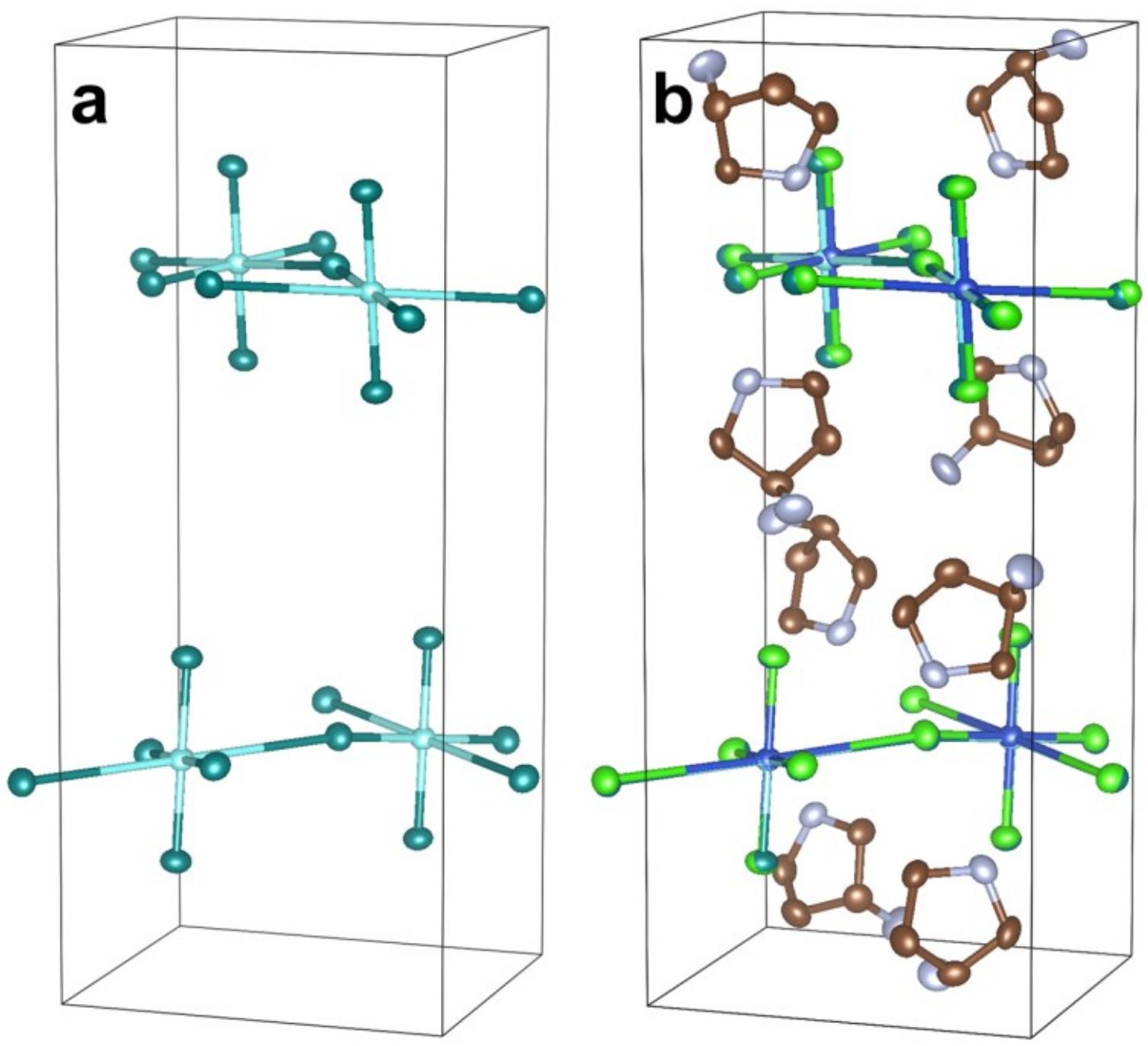

Figure S2. (a) Centrosymmetric inorganic framework of (R)-$(C_4H_9FN)_2CuCl_4$ (R)-fluoropyrrolidinium copper(II) output by ADDSYM, and **(b)** experimental (chiral) crystal structure rendered on top of centrosymmetric framework shown in (a). H atoms omitted in (b) for clarity.

3. Powder X-Ray Diffraction (PXRD) Pattern

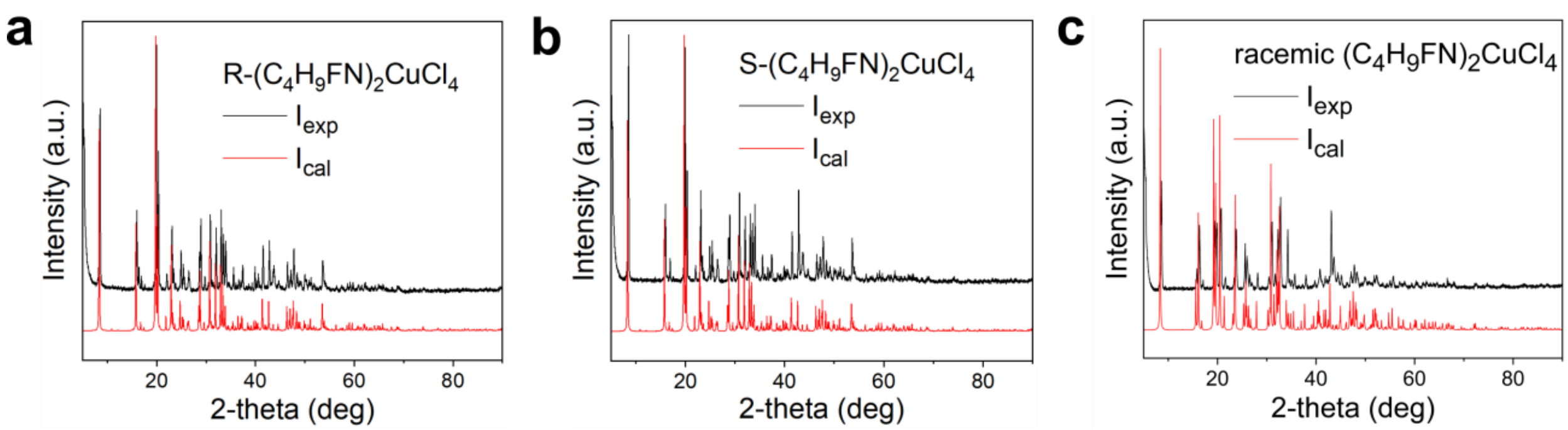

Figure S3. Experimental (I_{exp}) and calculated (I_{cal}) powder X-ray diffraction pattern of **(a)** (R)-($C_4H_9FN)_2CuCl_4$, **(b)** (S)-($C_4H_9FN)_2CuCl_4$, and **(c)** racemic ($C_4H_9FN)_2CuCl_4$ powders.

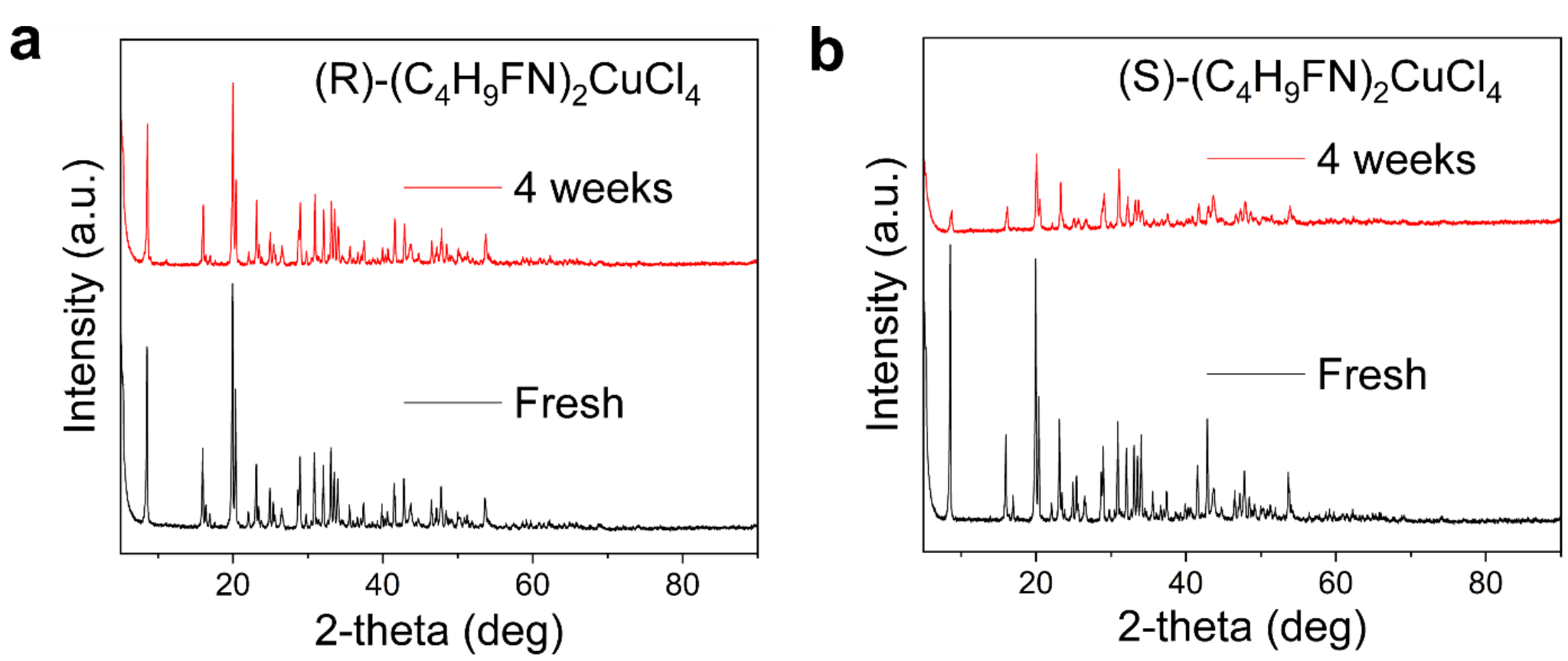

Figure S4. PXRD pattern measured for **(a)** (R)- and **(b)** (S)-($C_4H_9FN)_2CuCl_4$ to show their stability against ambient air exposure.

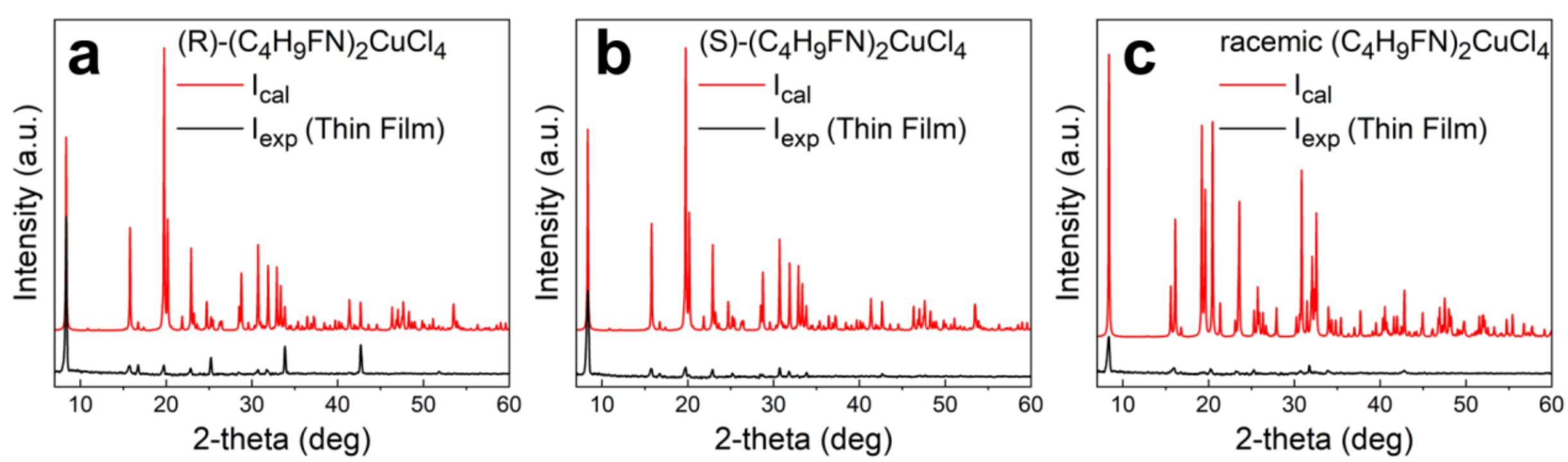

Figure S5. Experimental (I_{exp}) and calculated (I_{cal}) X-ray diffraction pattern of **(a)** (R)-($C_4H_9FN)_2CuCl_4$, **(b)** (S)-($C_4H_9FN)_2CuCl_4$, and **(c)** racemic ($C_4H_9FN)_2CuCl_4$ thin films.

4. Linear Dichroism and Linear Birefringence Correction to Circular Dichroism Spectra

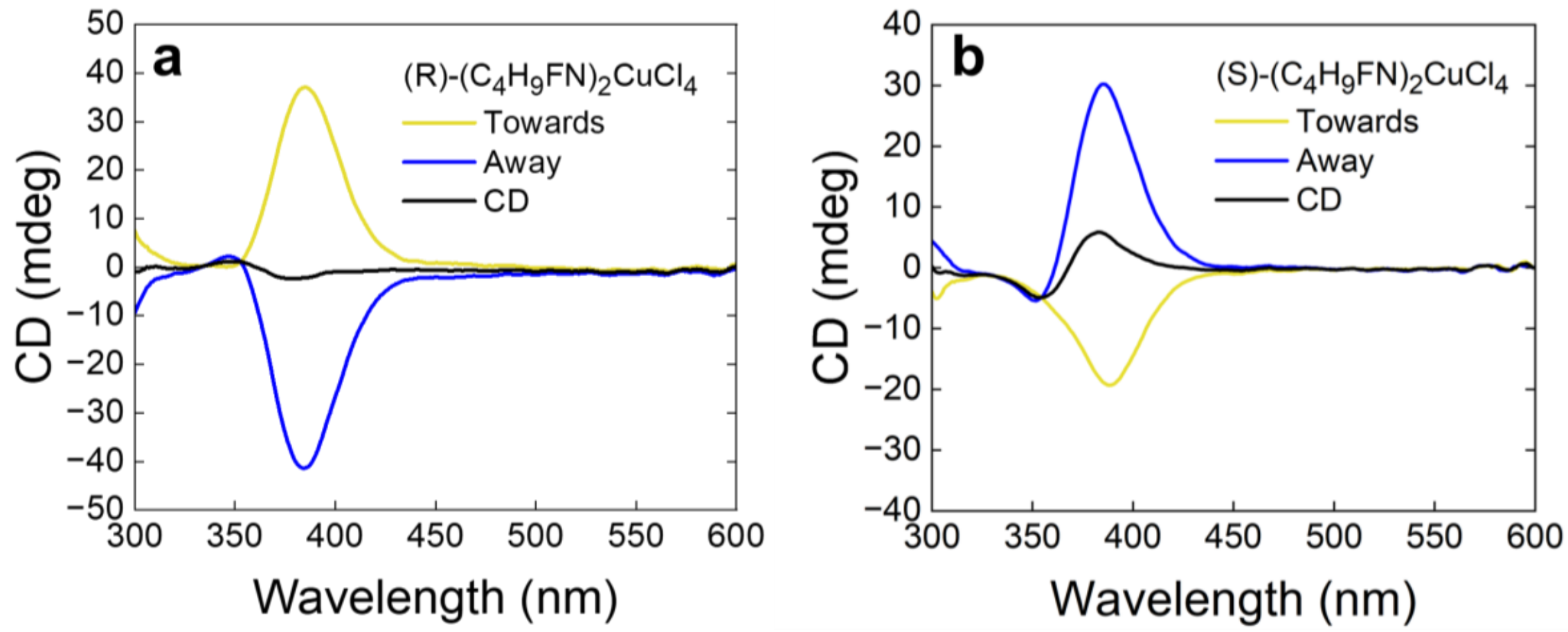

Figure S6. CD spectra of **(a)** (R)- and **(b)** (S)-$(C_4H_9FN)_2CuCl_4$ with (blue) film facing towards the detector, (yellow) away from the detector, and (black) true CD.

5. Plot of the Anisotropy Factor (g_{CD})

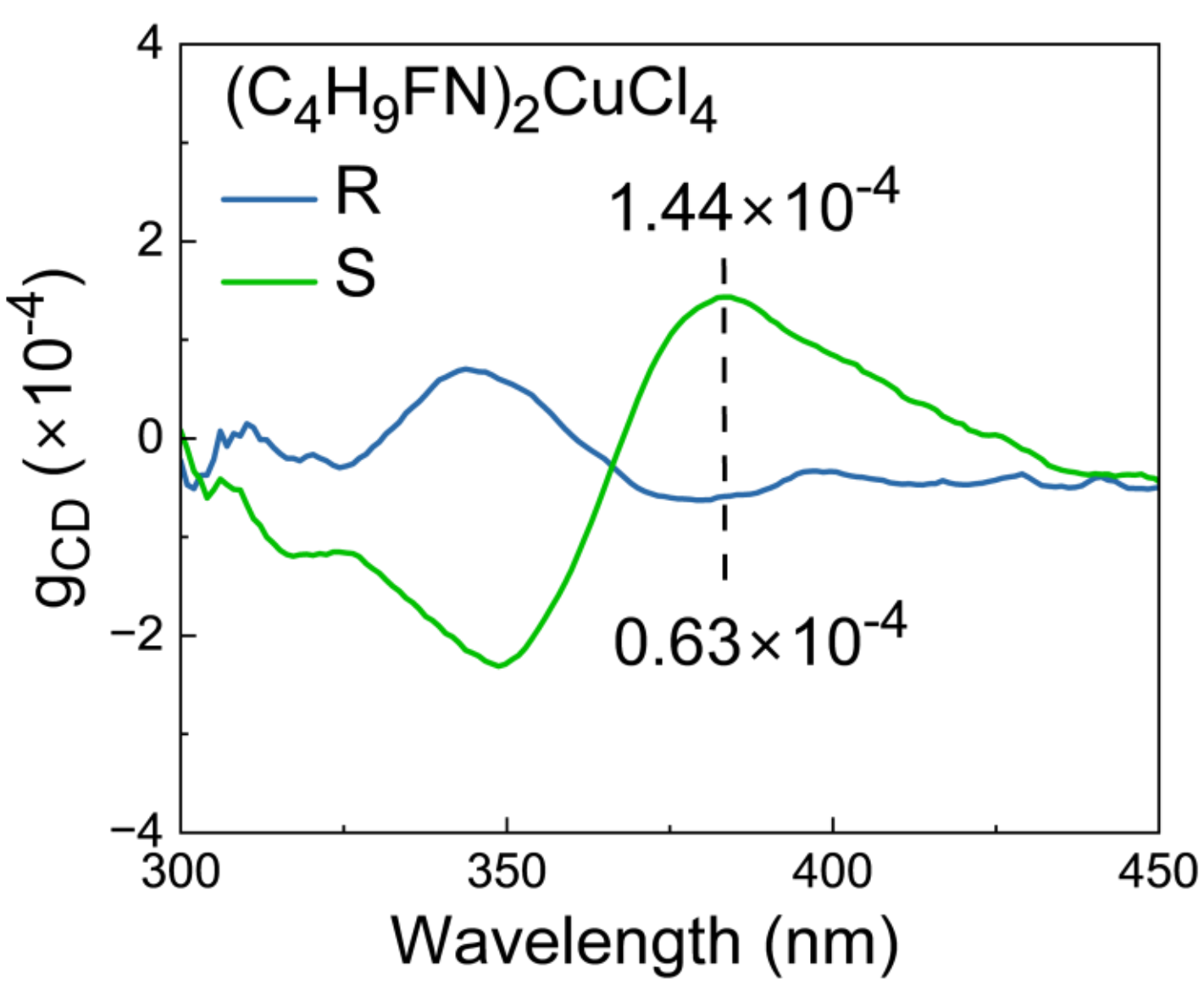

Figure S7. Plot of the anisotropy factor (g_{CD}) for (R)- and (S)-$(C_4H_9FN)_2CuCl_4$ thin films.

6. Additional Susceptibility Data on Powder Samples

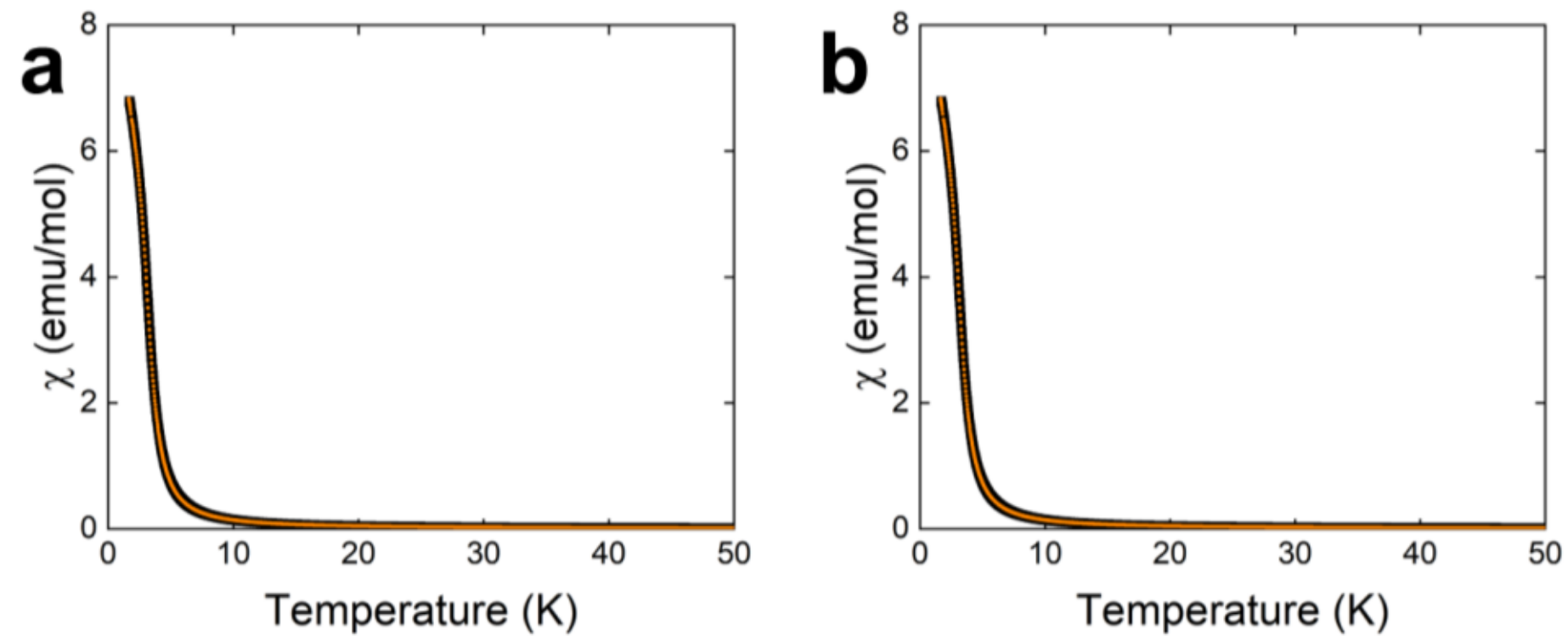

Figure S8. Plot of (black) zero-field cooled and (orange) field-cooled susceptibility data for **(a)** chiral $(C_4H_9FN)_2CuCl_4$ and **(b)** racemic $(C_4H_9FN)_2CuCl_4$ powder samples.

7. Heat Capacity Data

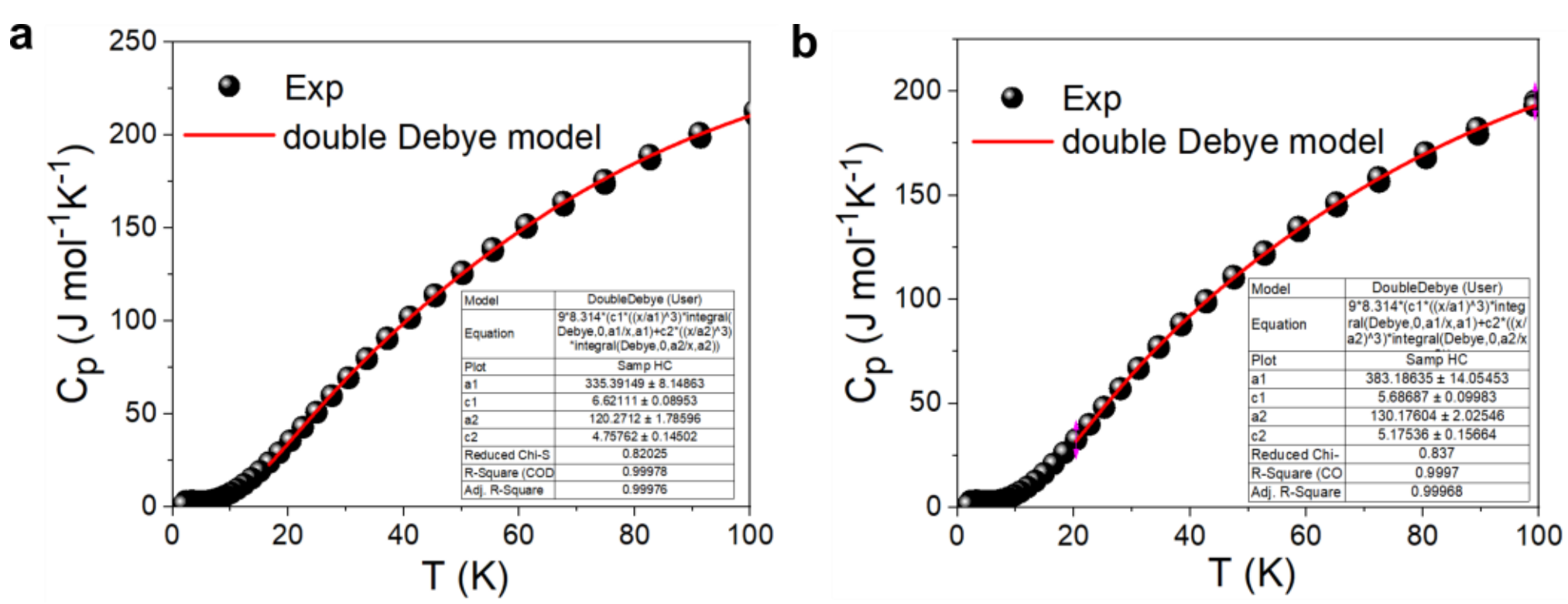

Figure S9. Plot of the heat capacity data vs. T for **(a)** chiral $(C_4H_9FN)_2CuCl_4$ and **(b)** racemic $(C_4H_9FN)_2CuCl_4$ single crystals.

8. AC Susceptibility Data

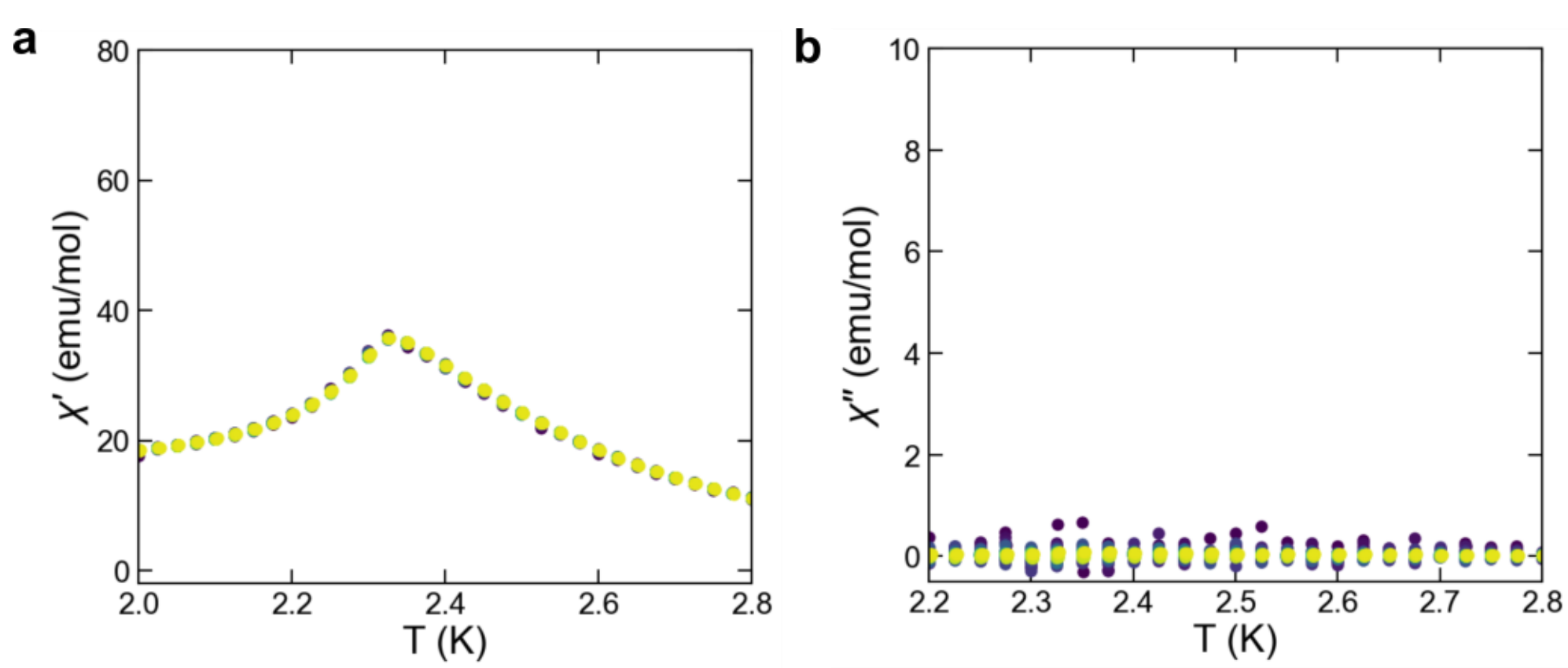

Figure S10. (a) In-phase (χ') component and **(b)** out-of-phase (χ'') component of AC susceptibility for (S)-$(C_4H_9FN)_2CuCl_4$.

9. Electronic Measurement Results

Figure S11a shows the current-voltage (I-V) measurement data using as-grown (R)-$(C_4H_9FN)_2CuCl_4$ crystals. Measured along the two-dimensional (2D) layer propagation direction (ab plane), a resistivity of ~10^4 Ω·cm is obtained. Perpendicular to the 2D layer propagation direction (c axis), we obtained a resistivity of ~10^7 Ω·cm. As expected, 2D metal halides show anisotropic electrical behaviors due to the insulating organic spacers that separate the metal-halide layers, resulting in a much greater resistivity when measured perpendicular to the layer direction. Similarly, the 2D Ruddlesden-Popper perovskite $(BA)_2CsPb_2Br_7$ (where BA is butylamine) has a resistivity of 2.22×10^9 Ω·cm and 9.87×10^{10} Ω·cm along the ab plane and c axis, respectively.[8] In addition, using the space-charge-limited-current (SCLC) method,[9] we were able to estimate the density of trap states and carrier mobility in (R)-$(C_4H_9FN)_2CuCl_4$ crystals from the trap-filled-limited (TFL) and Child regimes, respectively (Figure S11b). The density of trap states n_t is determined using the formula below,[9]

$$n_t = \frac{2\varepsilon\varepsilon_0}{eL^2} V_{TFL}$$

where ε (=13.02) is the dielectric constant, ε_0 is the vacuum permittivity, e is the electronic charge, L (=1 mm) is the crystal thickness, and V_{TFL} is the onset voltage of the TFL regime. The density of trap states is determined to be 4.0×10^{10} cm^{-3} for (R)-$(C_4H_9FN)_2CuCl_4$. This value is comparable to other metal halides (e.g., 2.77×10^{11} cm^{-3} for one-dimensional $CsCu_2I_3$, 3.33×10^{10} cm^{-3} for 2D $Rb_4Ag_2BiBr_9$.[10,11] For the charge carrier mobility, it is determined according to the Mott-Gurney law,[12]

$$\mu = \frac{8J_D L^3}{9\varepsilon\varepsilon_0 V^2}$$

where μ is the carrier mobility, J_D is the current density at bias voltage V and L is again the crystal thickness. The carrier mobility is calculated to be 0.45 cm^2/V-s for (R)-$(C_4H_9FN)_2CuCl_4$.

To study the photo-response characteristics of $(C_4H_9FN)_2CuCl_4$, we fabricated a prototype photodetector using a (R)-$(C_4H_9FN)_2CuCl_4$ crystal. The crystal was exposed continuously to 365 nm light illumination (Figure S11c). For the fabricated photodetector, we obtained an on/off ratio ($= \frac{I_{light\ on}}{I_{dark}}$, where I_{dark} is the current measured under dark environment with no light exposure) of ~1.55 (at detector bias +20 V). Although the on/off ratio obtained here for (R)-$(C_4H_9FN)_2CuCl_4$ is relatively low, it should be noted that the on/off ratio for photodetectors is highly dependent on the detector bias, light illumination intensity, and wavelength of the light used for illumination. We also noticed that the photocurrent decreases over the short measurement period. This decrease is potentially due to the ionic conductivity of metal halides, which has been commonly observed in TlBr, $MAPbI_{3-x}Cl_x$, and $CsPbCl_3$ semiconductors.[13–15]

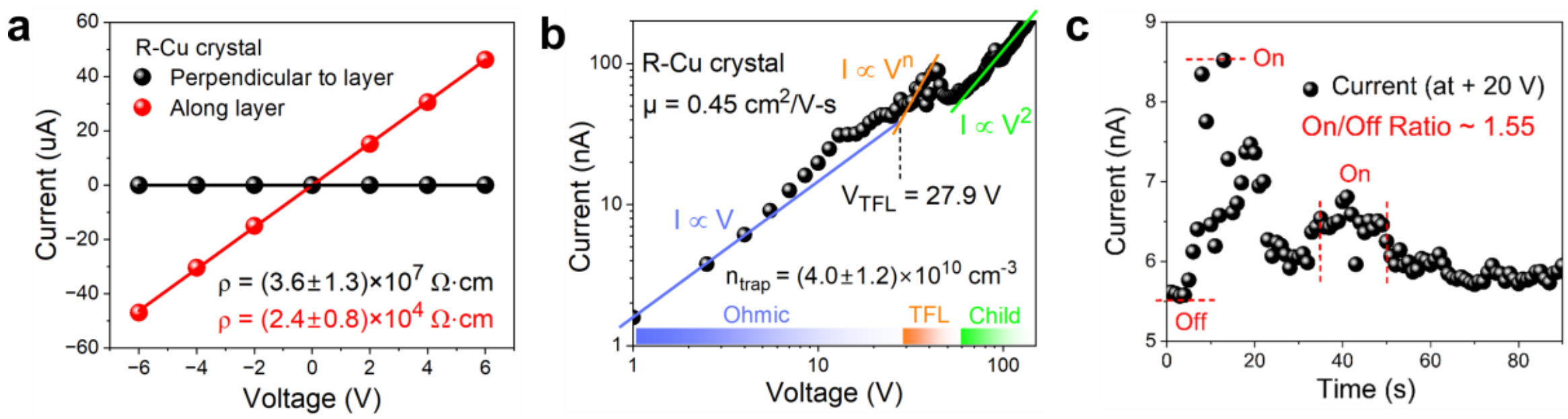

Figure S11. (a) I-V measurement using (R)-$(C_4H_9FN)_2CuCl_4$ crystal to determine the resistivity; **(b)** SCLC measurement data for an as-grown (R)-$(C_4H_9FN)_2CuCl_4$ crystal; **(c)** Response of (R)-$(C_4H_9FN)_2CuCl_4$ crystal to 365 nm light illumination. The fabricated photodetector was biased at +20 V.

10. Output from PLATON's CALC ADDSYM EXACT SHELX Command on (R)-$(C_4H_9FN)_2CuCl_4$

```
:: Current Graphics Settings: DISPLAY  = on ; DISPLAY  TYPE =  X11
:: ===(See HELP GRAPHICS)===: METAFILE = off; METAFILE TYPE =   PS

:: Data from: r-cu-300k_a.cif

:: Restricted CIF-File Format assumed (Automatic NOMOVE effective)

:: Default Global AUDIT TOOL Name: Olex2 1.5

:: Number of Valid CIF data_ ENTRIES =     1


:: Loaded Data Set: r-cu-300k

:: Extracted from CIF: r-cu-300k_a_sx.ins
:: Extracted from CIF: r-cu-300k_a_sx.hkl

::                         *** S.e.l.e.c.t.e.d  I.n.s.t.r.u.c.t.i.o.n.s ***
:: ************************ CALC ALL    - for an exhaustive geometry calculation
:: *        PLATON        * ORTEP      - for default labeled ORTEP-look-alike
:: *        ======        * VALID      - for a PLATON/checkCIF validation run
:: *    A Multipurpose    * LEPAGE     - to  check for higher metrical symmetry
:: *   Crystallographic   * ADDSYM     - for a check for MISsed SYMmetry
:: *         Tool         * NONSYM     - for a non-cryst. symm. check
:: *          --          * SOLV       - to  search for missed solvent areas
:: *(C) 1980-2026 A.L.Spek* SQUEEZE    - to  handle disordered solvents
:: *          --          * NEWMAN     - for NEWMAN-Projection Plots
:: *   version :  30426   * LIST RADII - for current atomic radii list
:: *   scratch :  400MB   * HELP       - for Available Instruction Information
:: ************************ PLUTON     - to  enter the PLUTON sub-program
::
>>INCLUDE CU CL
>>CALC ADDSYM EXACT SHELX 0.2 0.2 0.2 0.2

:: TITL r-cu-300k        P 21 21 21   R = 0.04
:: LAMBDA    0.71073
:: CELL     7.7559    8.7973    21.1758    90.000    90.000    90.000    1444.8
:: SPGR P 21 21 21    Chiral

- ADDSYM Search on ALL NON-H Atom Types [Max Allowed NonFit  0 % ]
- The  ADDSYM Search may be rerun for a choosen atom type
- Use LIST RADII for an overview of the atom types

- Number of Input Atoms Included in Search    5 (Unitcell   20)
```

```
- Density based on Input Atom Set = 1.773 g.cm-3 - Vol / Non-H atom = 21.2 Ang^3
- The Structure Implies the Following Symmetry Elements Subject to the Criteria:
- Criteria 0.20 Deg (Metric), 0.20 Ang (Rot), 0.20 Ang (Inv), 0.20 Ang (Transl)
- Pivot Atom Cu1

Symm.  Input  Reduced  (Ang)        (Deg)  %   AvrDev.(Ang)          Input Cell
Elem Cell_Row Cell_Row   d  Typ Dot Angle Fit  MaxDev.             x     y     z
--------------------------------------------------------------------------------
 c * [ 1 0 0] [ 1 0 0]  7.76  2  1     0  100   0.113  Through 0.494     0     0
                                    Cl3  -Cl1   0.193  Glide       0     0   1/2
 a * [ 0 1 0] [ 0-1 0]  8.80  2  1     0  100   0.093  Through     0 0.491     0
                                    Cl1  -Cl3   0.193  Glide     1/2     0     0
 b * [ 0 0 1] [ 0 0 1] 21.18  2  1     0  100   0.093  Through     0     0 0.498
                                    Cl1  -Cl3   0.193  Glide       0   1/2     0
-1 * ===================================  100   0.093  at      0.244 0.241 0.248
                                    Cl1  -Cl3   0.193

         T.R.A.N.S.F.O.R.M.A.T.I.O.N  M.A.T.R.I.X for CELL and HKL DATA
         ==============================================================
   Reduced->Convent         Input->Reduced       T = Input->Convent:    a' = T a
--------------------------------------------------------------------------------
(     0    -1     0 )   (     1     0     0 )   (     0     1     0 )     Det(T)
(     1     0     0 ) X (     0    -1     0 ) = (     1     0     0 )       =
(     0     0     1 )   (     0     0    -1 )   (     0     0    -1 )     1.000

Cell Lattice  a       b       c    Alpha   Beta  Gamma Volume CrystalSystem Laue
--------------------------------------------------------------------------------
Input   oP  7.756   8.797  21.176  90.00  90.00  90.00   1445 orthorhombic   mmm
Reduced  P  7.756   8.797  21.176  90.00  90.00  90.00   1445
Convent oP  8.797   7.756  21.176  90.00  90.00  90.00   1445 orthorhombic   mmm

          Conventional, New or Pseudo Symmetry
================================================================================

Space Group  H-M:  Pbca                                    Laue:  mmm
Space Group Hall: -P 2ac 2ab                     [Schoenflies: D2h^15]
Lattice Type: oP,  Centric, Orthorhombic, Multiplicity:   8( 4), No:  61

Non-Sohncke - No Absolute Structure

  Nr            ***** Symmetry Operation(s) *****

   1               X ,              Y ,              Z
   2         1/2 - X ,            - Y ,        1/2 + Z
   3         1/2 + X ,        1/2 - Y ,            - Z
   4             - X ,        1/2 + Y ,        1/2 - Z
   5             - X ,            - Y ,            - Z
   6         1/2 + X ,              Y ,        1/2 - Z
   7         1/2 - X ,        1/2 + Y ,              Z
   8               X ,        1/2 - Y ,        1/2 + Z

:: Origin Shifted to: 0.2410, 0.2444,-0.2484 after Transformation

::                      (  0.0000  1.0000  0.0000) ( -0.2410)
:: R/t for Coordinates  (  1.0000  0.0000  0.0000) ( -0.2444)
::                      (  0.0000  0.0000 -1.0000) (  0.2484)


:: - Symmetry Elements Preceded by an Asterisk are New and Indicate
::   Missed/Pseudosymmetry.
:: - Proposed Inversion or (Glide) Planes do NOT Apply
::   for Chiral Molecules.
:: - Glide Plane Codes are with Reference to the Input Cell !!

P! r-cu-300kP 21 21 21 oP=>oP 0.3 0.000 0.00 0.193 1.00   100% Pbca


:: An SPF-style file is written to be used for the cell transformation.

:: Rounding set to OFF
```

```
:: Create r-cu-300k_a_pl.spf
:: Resd  1, SOF 1.000, Z  4, Cl4 Cu

:: Moiety_Formula = Cl4 Cu
:: Sum_Formula    = Cl4 Cu
:: Formula_Weight =      205.35 [Note: Based on SHELXL2019 Atomic Weights]
:: Formula_Z      =           4
:: SpaceGroup_Z   =           4
:: Formula_Z'     =       1.000
:: mu(MoKa)       =       21.85 cm-1 =   2.185 mm-1
:: Res.Scat.      =         409 x 0.0001
:: Friedif_stat   =         474
:: Predicted Vol  =       520.4[     520.5] Ang**3, 298[300]K

:: Resd  MolVol     Formula          - (Kitaigorodskii Analytical Formula)
!K    1    94.6 A^3 Cl4 Cu

================================================================================
Summary and Remarks : N = NOTE, W = WARNING, E = ERROR
================================================================================
W: NOMOVE option used.
:: >>> WARNING: 'CONNECTED INPUT SET' is assumed to be TRUE
:: >>> The Network Analysis may be INCORRECT when this  assumption is FALSE
N: ADDSYM finds additional (pseudo)symmetry in the structure (please check!)
N: No-Hydrogen atoms in this structure
================================================================================


:: Input Xtal Data from File r-cu-300k_a.spf - Data Type SPF14

:: NORMAL END of PLATON :     8 Pages on FILE r-cu-300k_a.lis


:: Loaded Data Set: r-cu-300k        P 21 21 21   R = 0.04 New:Pbca

:: Transformation for x,y,z coordinates
:: (  0.0000  1.0000  0.0000) (x)     -0.2410
:: (  1.0000  0.0000  0.0000) (y) +   -0.2444
:: (  0.0000  0.0000 -1.0000) (z)      0.2484

:: PAR( 22) old value =    0.3000 new =    0.7000

:: PLA100-CELL-IN     7.7559    8.7973   21.1758   90.0000   90.0000   90.0000
:: PLA100-CELL-OUT    8.7973    7.7559   21.1758   90.0000   90.0000   90.0000

:: ATOM CL3     DELETED from INPUT STREAM,  0.03 Ang. From Cl1
:: ATOM CL3     at 0.03 Ang. from Cl1     - New Av Pos:  0.0019  0.4526 -0.1054
:: ATOM CL4     DELETED from INPUT STREAM,  0.04 Ang. From Cl2
:: ATOM CL4     at 0.04 Ang. from Cl2     - New Av Pos:  0.1627  0.7285 -0.0136
:: SHELXL HKLF Matrix:  0.00  1.00  0.00  1.00  0.00  0.00  0.00  0.00 -1.00

:: Coord. Cell:     8.797     7.756    21.176     90.00     90.00     90.00
:: Refln. Cell:     7.756     8.797    21.176     90.00     90.00     90.00

:: IPR(209) old value =         0 new =         1
:: Rounding set to OFF

:: TITL r-cu-300k        P 21 21 21   R = 0.04 New:Pbca
:: LAMBDA   0.71073
:: CELL     8.7973    7.7559   21.1758    90.000    90.000    90.000    1444.8
:: SPGR P b c a
:: Resd  1, SOF 1.000, Z  4, Cl4 Cu

:: Moiety_Formula = Cl4 Cu
:: Sum_Formula    = Cl4 Cu
:: Formula_Weight =      205.35 [Note: Based on SHELXL2019 Atomic Weights]
:: Formula_Z      =           4
:: SpaceGroup_Z   =           8
:: Formula_Z'     =       0.500
:: mu(MoKa)       =       21.85 cm-1 =   2.185 mm-1
:: Predicted Vol  =       520.4[     520.5] Ang**3, 298[300]K
```

```
Space Group  H-M:  Pbca                                    Laue:  mmm
Space Group Hall: -P 2ac 2ab                        [Schoenflies: D2h^15]
Lattice Type: oP,  Centric, Orthorhombic, Multiplicity:   8( 4), No:  61

Non-Sohncke - No Absolute Structure

  Nr           ***** Symmetry Operation(s) *****

   1               X ,              Y ,              Z
   2         1/2 - X ,            - Y ,        1/2 + Z
   3         1/2 + X ,        1/2 - Y ,            - Z
   4             - X ,        1/2 + Y ,        1/2 - Z
   5             - X ,            - Y ,            - Z
   6         1/2 + X ,              Y ,        1/2 - Z
   7         1/2 - X ,        1/2 + Y ,              Z
   8               X ,        1/2 - Y ,        1/2 + Z
SFAC  C   2.31000  20.84392   1.02000  10.20751   1.58860   0.56870   0.86500 =
         51.65125   0.21560   0.00330   0.00160    11.500   0.68000  12.01000
SFAC  H   0.49300  10.51091   0.32291  26.12573   0.14019   3.14236   0.04081 =
         57.79977   0.00304   0.00000   0.00000     0.624   0.35000   1.00800
SFAC Cl  11.46041   0.01040   7.19641   1.16620   6.25561  18.51942   1.64550 =
         47.77846  -9.55741   0.14840   0.15850   678.000   0.99000  35.45000
SFAC Cu  13.33801   3.58280   7.16761   0.24700   5.61581  11.39661   1.67350 =
         64.81267   1.19100   0.32010   1.26510  5180.000   1.52000  63.55000
SFAC  F   3.53920  10.28251   2.64120   4.29440   1.51700   0.26150   1.02430 =
         26.14763   0.27760   0.01710   0.01030    51.500   0.64000  19.00000
SFAC  N  12.21261   0.00570   3.13220   9.89331   2.01250  28.99754   1.16630 =
          0.58260 -11.52901   0.00610   0.00330    19.600   0.68000  14.01000

:: Resd  MolVol     Formula          - (Kitaigorodskii Analytical Formula)
!K    1    94.6 A^3 Cl4 Cu

================================================================================
Summary and Remarks : N = NOTE, W = WARNING, E = ERROR
================================================================================
W: NOMOVE option used.
:: >>> WARNING: 'CONNECTED INPUT SET' is assumed to be TRUE
:: >>> The Network Analysis may be INCORRECT when this  assumption is FALSE
N: ADDSYM finds additional (pseudo)symmetry in the structure (please check!)
N: No-Hydrogen atoms in this structure
W: Low density (check!) of ....................................   0.944 gcm-3
N: Input Data following TRNS [e.g. (CELL/CESD)/SPGR/COORDS/UIJ)] have been
    transformed according to the specified Cell Transformation Matrix:
                       0.00000   1.00000   0.00000
                       1.00000   0.00000   0.00000
                       0.00000   0.00000  -1.00000
N: Input data (COORDINATES) have been transformed
    according to additive coordinate shift vector:
                      -0.24104  -0.24444   0.24842
N: Number of deleted ATOMS from input stream ............................   2
N: Number of Ignored Lines on INPUT .....................................   6
          of which blank in column 1 ....................................   0
================================================================================


:: Input Xtal Data from File r-cu-300k_a.spf - Data Type SPF

:: NORMAL END of PLATON :    16 Pages on FILE r-cu-300k_a.lis

:: SHELXL   res on :r-cu-300k_a_pl.res
```

Supporting Information References

(1) Sheldrick, G. M. SHELXT – Integrated Space-Group and Crystal-Structure Determination. *Acta Crystallogr. Sect. Found. Adv.* **2015**, *71* (1), 3–8.
(2) Sheldrick, G. M. Crystal Structure Refinement with SHELXL. *Acta Crystallogr. Sect. C Struct. Chem.* **2015**, *71* (1), 3–8.
(3) Dolomanov, O. V.; Bourhis, L. J.; Gildea, R. J.; Howard, J. A. K.; Puschmann, H. OLEX2 : A Complete Structure Solution, Refinement and Analysis Program. *J. Appl. Crystallogr.* **2009**, *42* (2), 339–341.
(4) Chen, G. C.; Yang, J. T. Two-Point Calibration of Circular Dichrometer with d-10-Camphorsulfonic Acid. *Anal. Lett.* **1977**, *10* (14), 1195–1207.
(5) Mazzeo, G.; Fusè, M.; Abbate, S.; Zagami, R.; Angela Castriciano, M.; Romeo, A.; Monsù Scolaro, L.; Longhi, G. Tuning Linear Dichroism/Birefringence (LDLB) Contributions in VCD Spectra of Tartaric Acid-TPPS 4 Porphyrin Films. *Phys. Chem. Chem. Phys.* **2025**, *27* (43), 23245–23256.
(6) Vázquez, J. T. Features of Electronic Circular Dichroism and Tips for Its Use in Determining Absolute Configuration. *Tetrahedron Asymmetry* **2017**, *28* (10), 1199–1211.
(7) Merten, C.; Kowalik, T.; Hartwig, A. Vibrational Circular Dichroism Spectroscopy of Solid Polymer Films: Effects of Sample Orientation. *Appl. Spectrosc.* **2008**, *62* (8), 901–905.
(8) Xiao, B.; Sun, Q.; Wang, F.; Wang, S.; Zhang, B.-B.; Wang, J.; Jie, W.; Sellin, P.; Xu, Y. Towards Superior X-Ray Detection Performance of Two-Dimensional Halide Perovskite Crystals by Adjusting the Anisotropic Transport Behavior. *J. Mater. Chem. A* **2021**, *9* (22), 13209–13219.
(9) Bube, R. H. Trap Density Determination by Space-Charge-Limited Currents. *J. Appl. Phys.* **1962**, *33* (5), 1733–1737.
(10) Han, S.; Quan, J.; Wang, D.; Li, H.; Liu, X.; Xu, J.; Zhang, Y.; Li, Z.; Wu, L.; Fang, X. Anisotropic Growth of Centimeter-Size CsCu2I3 Single Crystals with Ultra-Low Trap Density for Aspect-Ratio-Dependent Photodetectors. *Adv. Sci.* **2023**, *10* (7), 2206417.
(11) Zhang, Z.; Ma, Y.-Z.; Thomas, L.; Gofryk, K.; Saparov, B. Physical Properties of Candidate X-Ray Detector Material Rb4Ag2BiBr9. *Cryst. Growth Des.* **2022**, *22* (2), 1066–1072.
(12) de Boer, R. W. I.; Jochemsen, M.; Klapwijk, T. M.; Morpurgo, A. F.; Niemax, J.; Tripathi, A. K.; Pflaum, J. Space Charge Limited Transport and Time of Flight Measurements in Tetracene Single Crystals: A Comparative Study. *J. Appl. Phys.* **2004**, *95* (3), 1196–1202.
(13) Secco, R. A.; Secco, E. A.; Chen, Q. Defects and Ionic Conductivity in TlCl, TlBr, and TlI at High Pressure and Temperature. *J. Solid State Chem.* **1998**, *141* (2), 462–465.
(14) Mizusaki, J.; Arai, K.; Fueki, K. Ionic Conduction of the Perovskite-Type Halides. *Solid State Ion.* **1983**, *11* (3), 203–211.
(15) Głowienka, D.; Miruszewski, T.; Szmytkowski, J. The Domination of Ionic Conductivity in Tetragonal Phase of the Organometal Halide Perovskite CH3NH3PbI3-xClx. *Solid State Sci.* **2018**, *82*, 19–23.
